# Considerations for large building water quality after extended stagnation

**DOI:** 10.1002/aws2.1186

**Published:** 2020-08-06

**Authors:** Caitlin R. Proctor, William J. Rhoads, Tim Keane, Maryam Salehi, Kerry Hamilton, Kelsey J. Pieper, David M. Cwiertny, Michele Prévost, Andrew J. Whelton

**Affiliations:** ^1^ Division of Environmental and Ecological Engineering, Lyles School of Civil Engineering, Weldon School of Biomedical Engineering, School of Materials Engineering Purdue University West Lafayette Indiana; ^2^ Department of Civil and Environmental Engineering Virginia Tech Blacksburg Virginia; ^3^ Legionella Risk Management, Inc. Chalfont Pennsylvania; ^4^ Department of Civil Engineering University of Memphis Memphis Tennessee; ^5^ School of Sustainable Engineering and the Built Environment Arizona State University Tempe Arizona; ^6^ Department of Civil and Environmental Engineering Northeastern University Boston Massachusetts; ^7^ Department of Civil and Environmental Engineering, Seamans Center for the Engineering Arts and Sciences University of Iowa Iowa City Iowa; ^8^ Center for Health Effects of Environmental Contamination University of Iowa Iowa City Iowa; ^9^ Public Policy Center University of Iowa Iowa City Iowa; ^10^ Civil, Geological and Mining Engineering Polytechnique Montreal Montréal Québec Canada; ^11^ Lyles School of Civil Engineering, Division of Environmental and Ecological Engineering Purdue University West Lafayette Indiana

**Keywords:** advisory, building, coronavirus, COVID‐19, customer, disaster, disinfection, flushing, health risk, plumbing, recommissioning, SARS, SARS‐CoV‐2, stagnation, water quality

## Abstract

The unprecedented number of building closures related to the coronavirus disease (COVID‐19) pandemic is concerning because water stagnation will occur in many buildings that do not have water management plans in place. Stagnant water can have chemical and microbiological contaminants that pose potential health risks to occupants. Health officials, building owners, utilities, and other entities are rapidly developing guidance to address this issue, but the scope, applicability, and details included in the guidance vary widely. To provide a primer of large building water system preventative and remedial strategies, peer‐reviewed, government, industry, and nonprofit literature relevant to water stagnation and decontamination practices for plumbing was synthesized. Preventative practices to help avoid the need for recommissioning (e.g., routine flushing) and specific actions, challenges, and limitations associated with recommissioning were identified and characterized. Considerations for worker and occupant safety were also indicated. The intended audience of this work includes organizations developing guidance.

## 1 INTRODUCTION

In 2020, the global pandemic caused by novel coronavirus (SARS‐CoV‐2) disease (COVID‐19) prompted “stay‐at‐home” orders all over the world that closed or reduced occupancy in many nonessential businesses and other buildings (e.g., education, event, worship, recreation, office, and retail buildings) (Jiang, 2020; Lee, 2020a). With more than 5.6 million commercial buildings in the United States alone (CBECS 2012, 2015), the orders significantly altered drinking water demand patterns at both the water distribution and building system levels (American Water Works Association and Association of Metropolitan Water Utilities, 2020). Specifically, many buildings experienced reduced water use, causing increased water stagnation time (i.e., water age). This is problematic as stagnation has been associated with the degradation of water quality in routine settings (on a time scale of hours to days) at the building (Bédard, Laferrière, Déziel, & Prévost, 2018; Elfland, Paolo, & Marc, 2010; Lytle & Liggett, 2016; Nguyen, Elfland, & Edwards, 2012; Rhoads, Chamber, Pearce, & Edwards, 2015; Rhoads, Pearce, Pruden, & Edwards, 2015; Rhoads, Pruden, & Edwards, 2016; Salehi et al., 2018; Salehi et al., 2020) and water distribution system levels (American Water Works Association, 2002; Arnold & Edwards, 2012; AWWA, 2009; Brandt et al., 2005; Dias, Besner, & Prévost, 2017; Walksi et al., 2003), and can result in the presence of harmful chemicals (e.g., lead, copper) or harmful organisms (e.g., *Legionella pneumophila*) in water.

Water quality issues can be prevented or addressed with remedial actions, but the actions needed depend on the conditions of stagnation and on many site‐specific factors. Limited information is available regarding water quality impacts caused by extended stagnation and the effectiveness of plumbing remediation actions. Despite this lack of information, government agencies, water utilities, and private companies rapidly developed guidance to address the widespread building water system closures during the COVID‐19 pandemic. Some guidance documents have been cited in this paper, but many are emerging weekly (City of Durham, n.d.; American Water, 2020; Arkansas Department of Health Engineering, 2020; CDC, 2020; Connecticut Department of Public Health, 2020; Demarco, 2020; ESGL, 2020; ESPRI, 2020; ESPRI, AH Environmental Consultants, et al., 2020; Indiana Department of Environmental Management, 2020; Indiana State Department of Health, 2020; Ireland HSA, 2020; Minnesota Department of Health, 2020; New Zealand Ministry of Business and Environment, 2020; New Zealand Ministry of Health, 2020; Ohio Environmental Protection Agency and Ohio Department of Health, 2020; Oklahoma Department of Environmental Quality, 2020; Oregon Health Agency Public Health Division, 2020; PHE, 2020; PSPC, 2020; Public Health Madison & Dane County, 2020; Vancouver Coastal Health, 2020; Washington State Department of Health, 2020). This review provides an understanding of the challenges, current practices, and knowledge gaps for maintaining building water systems and restoring building water systems to baseline conditions after extended periods of no or limited water use. This review is not meant to explicitly serve as a step‐by‐step procedure; rather, it serves as a foundation for the development of step‐by‐step guidance. The intended audience of this review includes public health officials and other entities that are developing guidance. This may also be of interest to plumbing engineers and building owners who must consider many issues when implementing guidance, as well as to utilities to help coordinate their efforts with their customers. Guidance developed to address COVID‐19 pandemic stagnation or other prolonged stagnation events should address these considerations.

## 2 APPROACH

The authors reviewed literature regarding (a) water quality deterioration associated with shorter stagnation periods (hours to days) and parallel situations, (b) water quality in large buildings, (c) disease outbreaks associated with plumbing, and (d) plumbing decontamination practices. The authors also referenced guidance documents that may inform building owner responses to stagnation (Table S1), including plumbing codes, standards, guidance documents from various authorities, and recommendations from related professional organizations (ADEQ, 2015; AIHA, 2020; ASHRAE, 2000; ASHRAE Standards Committee, 2018; AWWA, 1992, 2014; CDC, 2020; Demarco, 2020; ESGL, 2020; ESPRI, AH Environmental Consultants, et al., 2020; IAPMO, 2018; International Code Council (ICC), 2018; OSHA, n.d.; PHE, 2020; US EPA Region 8, 2020; USEPA, 2013; WHO, 2011). Specific parallel situations included: (a) seasonal public water systems (e.g., campgrounds, fair grounds); (b) ski resort/snowbird communities with 80% reduction in water use in off‐seasons (Hasit, Anderson, Parolari, Rockaway, & French, 2006); (c) buildings unoccupied between owners; (d) athletic or other event centers, schools, and dormitories that have lower‐than‐design capacity water use seasonally; and (e) water shutoffs (e.g., due to nonpayment) that last weeks to months (Food & Water Watch, 2018; Kurth, 2019; Swain, McKinney, & Susskind, 2020). Finally, the authors' own first‐hand experiences assisting building owners who must decontaminate and restart plumbing after nonuse; conducting plumbing‐related disease outbreak investigations; and answering questions received from local, state, and federal agencies and nonprofit organizations about policy were considered.

This paper focuses on large buildings and campuses closed in spring 2020 to promote physical distancing (also known as social distancing). In this paper, the term building “recommissioning” is used to refer to the reopening of buildings after extended closures and focuses on restoring water quality to baseline conditions. Recommissioning here should not be confused with the recommissioning process featuring water audits and subsequent changes made to increase water and energy efficiency in buildings (e.g., changing toilets and fixtures to low‐flow) (Natural Resources Canada, 2018). The focus of this paper is the hot and cold water systems. The authors did not consider other necessary actions unrelated to water quality (e.g., air quality, heating systems) or for other building water systems (e.g., cooling towers), although these likely need attention. Other considerations will need to be taken for alternative building types (e.g., water shutoffs impacting 15 million people due to nonpayment (Food & Water Watch, 2018)). The repurposing of buildings or reopening of medical facilities to expand capacity also received attention during the COVID‐19 pandemic, and while these facilities face similar issues as those described here, medical facilities have additional considerations not included in this paper.

## 3 RESULTS AND DISCUSSION

### 3.1 Stagnation in large buildings

There are several concerns for water quality that are common in complex large building water systems, which can be made worse by periods of no or low water use (Gupta & Thawari, 2016; Julien et al., 2020; Lipphaus et al., 2014). It is necessary to understand these reactions, the associated health risks, and complexity and variability of building water systems when considering how to maintain water quality. The plumbing and water quality for each building will be unique.

**Figure 1:**
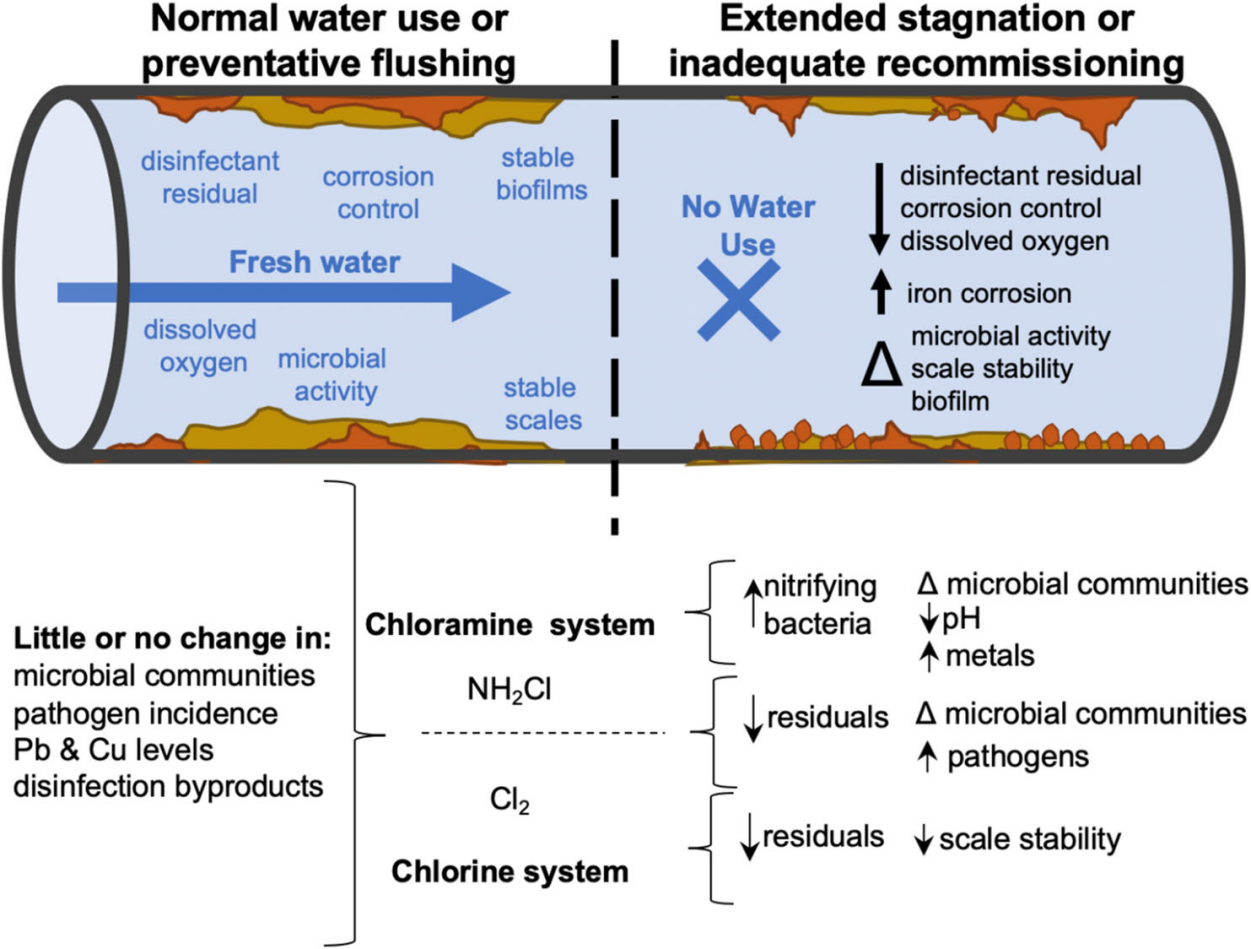
Potential chemical and microbial water quality impacts associated with prolonged stagnation in chlorine (Cl_2_) and chloramine‐based (NH_2_Cl) disinfectant drinking water systems

Reactions occurring during stagnation (Figure 1) include: (a) loss of disinfectant residual and decreased disinfectant residual stability; (b) decreased effectiveness of corrosion control measures; (c) microbial growth; and (d) other issues such as taste, odor, and disinfection byproduct formation. Microbial issues include nitrification, growth of harmful organisms (e.g., *Legionella pneumophila*, *Pseudomonas aeruginosa*, nontuberculous mycobacteria, others), and changes in microbial communities. These reactions, most of which have only been studied on a relatively short time scale, are described in SI‐1‐Stagnation Issues. It is currently unknown how these reactions may be impacted by long‐term stagnation on the order of weeks or months (e.g., growth reaching a carrying capacity with limited introduction of new nutrients).

Health risks from these reactions can be associated with the ingestion of, inhalation/aspiration of, or contact with contaminated water. Lead, copper, and other metals can leach from pipes or scales that may become unstable during long periods of stagnation. Increased growth of opportunistic pathogens such as *L. pneumophila* can also occur. Many such organisms cause pulmonary disease (e.g., Legionnaires' disease, Pontiac fever). Certain populations are at higher risk of adverse effects (e.g., children for lead exposure, immunocompromised persons for Legionnaire's disease). COVID‐19 patients may also be at risk for coinfection with *L. pneumophila* (Xing et al., 2020).

**Figure 2:**
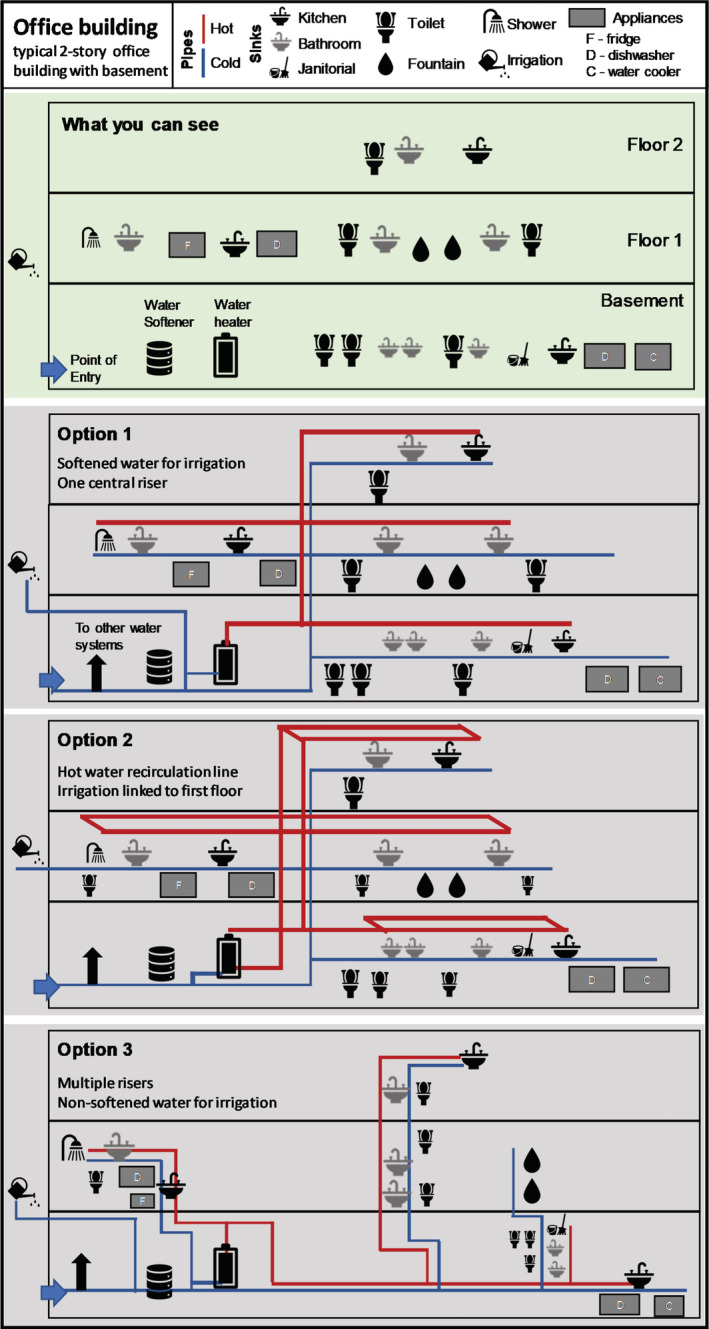
Building plumbing schematic. *Top*: what occupants can see; *Option 1*: Traditional trunk‐and‐branch; *Option 2*: trunk‐and‐branch with headers for every flow; *Option 3*: trunk‐and‐branch with multiple risers

Building water systems comprise all the piping, equipment, treatment devices, fixtures, and appliances associated with providing water from the service line point of entry (POE) (i.e., where water enters the building) to the point of use (POU). The number and type of plumbing components and materials encountered (Table 1) will depend on the building's water source, design (Figures 2 and S1), and water use applications. For example, water of various qualities (e.g., softened for drinking and unsoftened for handwashing) or temperatures (e.g., cold, multiple hot systems) might have their own parallel piping system through a building. Given the variability and complexity of plumbing, it is difficult to make generalizations.

**Table 1:** Types of building plumbing components

Components	Description
Water source	Municipal water, onsite well, treated surface water, rainwater.
Service line	Pipe system that carries water from the source to the building water system. Service line materials are variable and may or may not be the same as indoor pipes.
Safety devices including valves	Pressure relief valve, pressure reduction value, isolation valve, mixing valve, thermostatic mixing valves, backflow prevention device, water hammer arrestors. Materials can include aluminum, brass, copper, lead, plastic, and stainless steel.
Water treatment devices	Filter, strainer, water softener, chemical addition equipment for disinfection and corrosion control.
Water service and distribution piping and faucet connectors	Various material types have been used, including acrylonitrile butadiene styrene (ABS), brass, cast iron (CI), chlorinated polyvinyl chloride (CPVC), copper, crosslinked polyethylene (PEX), ductile iron (DI), high density polyethylene (HDPE), lead, lead lined steel, multilayer pipes, polyethylene raised temperature (PERT), polypropylene (PP), unplasticized polyvinyl chloride (uPVC), polyvinylidene fluoride (PVDF), black steel, stainless steel.
Hot water recirculation system	Hot water is pumped through primary and secondary water heater loops, which serve different building zones to reduce the delivery time of hot water. These have to be hydraulically balanced. Equipment includes master mixing valves, local mixing valves, flow‐balancing valves, pressure‐reducing valves, hot water return pumps, and water heaters. Multiple temperature loops may exist. Operation of pumps may be intermittent in some systems.
Fixtures and fixture fittings	Aerator, air washers, atomizers, bathtub, bidet, decorative fountains, dishwasher, drinking fountain, eyewash stations, manual faucet, electronic faucet, faucet flow restrictors, hoses, point‐of‐use mixing valves, hot tubs, humidifiers, ice machines, misters, shower head, shower wand, sink, tub spout, toilet, urinal, washbasin
Pumps	Pumps are often used for pressure boosting within the building (i.e., for multistory buildings) where water pressure entering the building is not adequate for water use at distal locations. Pumps are also used for hot water recirculation systems.
Tanks	Standard water heater, pressure tanks, on‐demand water heater, hydropneumatic tanks, cold water supply storage tank. Water heaters can contain Mg or Al sacrificial anodes and plastic dip tubes.
Point‐of‐use devices	On‐faucet treatment system, under sink treatment system.

*Note*: ASHRAE 188 defines the delivery system for hot and cold water as the “potable” water system (ASHRAE Standards Committee, 2018), and it is sometimes referred to as “domestic” water. Some of the components contain both metal and plastic subparts. These include gaskets; polysulfone or PEX dip tubes; and liners and coatings such as glass, ceramic, epoxy, polyurethane, polyurea, and fiberglass. Gaskets may be ethylene propylene diene monomer (EPDM) (sulfur or peroxide crosslinked), butyl rubber (BR), natural rubber (NBR), neoprene, styrene butadiene rubber (SBR), and synthetic rubber.

### 3.2 Water management practices under normal use

Normal building operation can often result in stagnation (e.g., offices over the weekend, unused hotel rooms), and the reactions described in Sections 3.1 and SI‐1 (a continued discussion on reactions occurring during stagnation) continuously occur at highly variable rates. Green buildings may be especially impacted by stagnation as they are designed for lower water use without substantially changing plumbing design (Rhoads et al., 2016; Rhoads, Pearce, et al., 2015; Salehi et al., 2020). To manage the water quality issues that occur even with normal use, some buildings (e.g., healthcare) are required to have building water management plans (BWMPs) (ASHRAE Standards Committee, 2018; CMS, 2017; VHA, 2014). However, in a small survey, nearly 60% of commercial building respondents (*n* = 29) had not heard of building water management plans (Masters, Clancy, Villegas, LeChevallier, & Bukhari, 2018).

Building water management plans help guide prevention and response to water quality issues, especially opportunistic pathogen growth. The management of building water ultimately requires a diversity of stakeholders to (a) supply water; (b) design, construct, operate, and maintain the system; (c) recommend and implement public health interventions; and (d) enforce applicable codes and regulations, which vary widely by state. Each category has individual building components that require a range of stakeholder involvement and may require one or more external vendors/suppliers to coordinate and manage. Development of BWMPs may also be considered in response to COVID‐19‐related stagnation (Table 2, details in Table S2) (CDC, 2020; ESPRI, AH Environmental Consultants, et al., 2020); however, the full implementation of BWMPs can take years and require substantial resources, and these documents do not necessarily address long periods of low or no water use explicitly. Building owners and creators of COVID‐19‐related plumbing guidance may want to instead focus on the most important aspects related to water quality and on quickly achievable goals. This includes keeping water fresh and thermal regulation (i.e., keeping cold water cold and hot water hot). Resources are readily available to aid in the development of these plans (ASHRAE, 2000; ASHRAE Standards Committee, 2018; CDC, 2017a).

**Table 2:** Guidance developed since COVID‐19 for building water management

Listed in order of Most recent date issued	Specific health risks explicitly identified	Action during building closures	Actions suggested prior to building use						
			Inspection	Flushing (amount, speed)	Other cleaning	Shock disinfection	Other step	Worker safety mentioned	Testing
Expert report (this study) & Key messages in the supplementary information	*Legionella*, *Mycobacteria*, *Pseudomonas aeruginosa*, free‐living amoeba, High lead and copper concentrations, Disinfection byproducts	Flush at least weekly, Consult public health authority, Maintain temperatures, Advise against draining	Several potential actions described. Consult public health authority					YES—aerosols with pathogens, shock disinfection water, and scalding	Consult public health authority
Nations and organizations with multiple nations represented									
ESGLI (2020): Guidance for Managing *Legionella* in Building Water Systems during the COVID‐19 Pandemic (ESGL, 2020); PHE (2020): COVID‐19 and Food Water and Environmental Microbiology Services (PHE, 2020)	*Legionella*	Inspection, flush weekly, Maintain temperatures, Monitor disinfectant residual, Shock disinfection before shutdown	NO	YES—Limited instruction	NOT SPECIFIC	YES—FULL	YES—Refill water heaters	YES— SCALDING	Temperature Biocide *Legionella*
CDC (2020): Guidance for Building Water Systems (CDC, 2020)	*Legionella,* Biofilm‐associated bacteria	Water management plan	YES—Slime only	YES	SOME SPECIFIC ACTIONS	YES— PARTIAL	YES—Water management plan	NO	Disinfectant residual
Ireland HSA (2020) Control of Legionella Bacteria During and After the COVID‐19 Pandemic (Ireland HSA, 2020)	*Legionella*	Training employees, *Legionella* control plan, Flushing (weekly), Risk assessment, DO NOT drain	Guidance references these processes but does not give instructions.						
Canada PSPC (2020): Building Water System Minimum Requirements (PSPC, 2020)	*Legionella,* Lead	Flush every 3 days to 1 week, (with detail instructions) Maintain a log Post signage	NO	YES—Detailed instructions depending on use condition	MANY SPECIFIC ACTIONS	NO	YES—Provide alternative water until proven safe	YES—Mentions personal protective equipment (PPE) and opening outlets slowly	Microbiology
New Zealand Ministry of Business and Environment (2020): Ensuring the Safety of Building Water System Post COVID 19 Lockdown (New Zealand Ministry of Business and Environment, 2020)	*Legionella* Microorganisms/pathogens Heavy metals	NO	YES—Floor drains	YES—Detail instructions	NO	NO	NO	NO	NO
New Zealand Ministry of Health (2020): COVID‐19 Drinking Water Advice: Returning to Normal Service (New Zealand Ministry of Health, 2020)	NO	NO	YES—Detailed for private water supply; or communicate with municipal supplier	YES—“Until appears normal” with some other instructions	NO	NO	NO	NO	NO
U.S. State, Canadian Province, and local agencies									
Connecticut Department of Public Health (2020): Building Water System Return to Service Guidance (Connecticut Department of Public Health, 2020)	Biofilm/*Legionella* growth, corrosion resulting in discolored water, odor, lead and/or copper release, and disinfection byproduct formation	NO	YES—Long list of items	YES—Detailed instructions including water volume calculation	MANY SPECIFIC ACTIONS	YES—With certain conditions	YES—Ongoing flushing 1/day for 12 weeks	YES—Mentions PPE and opening outlets slowly	Total coliform (all buildings) *Legionella* (some buildings)
Washington Department of Health (2020) COVID‐19 Guidance for Legionella and Building Water System Closures [version 2, April 30, 2020] [36]^a^	*Legionella*, *Mycobacterium avium*, lead	PREVENTATIVE AND REMEDIAL FLUSHING	NO	YES	SOME SPECIFIC ACTIONS	YES	YES	YES	*Legionella*
Indiana Department of Environmental Management (2020): IDEM Guidance for Flushing Water Systems (Indiana Department of Environmental Management, 2020)	*Legionella*, corrosion issues	NO	NO	YES—Detail instructions including time‐based	SOME SPECIFIC ACTIONS	NO	NO	YES—Mentions PPE	NO
Indiana State Department of Health (2020): Building Water System Startup Guidance (Indiana State Department of Health, 2020)	Lead, copper, *Legionella*	NO	YES—Long list of items	YES—Detail instructions including time‐based	NO	NO	YES—More actions depending on test results; Centers for Disease Control and Prevention guidance if municipal water source	NO	“Bacteriology” Metals (lead, copper)
Oklahoma Department of Environmental Quality (2020) Water Quality Recommendations for Opening Closed or Less Frequently Used Buildings (Oklahoma Department of Environmental Quality, 2020)	Lead, copper, disinfection byproducts, *legionella*, biofilm‐associated bacteria	Create water management plan, maintain water heater, flushing,	YES—Maintain system	YES—Some detail including flushing in stages	SOME SPECIFIC ACTIONS	NO	YES—Contact utility	NO	Temperature pH Disinfectant
Oregon Health Agency Public Health Division (2020) Guidance for Reopening Building Water Systems after Prolonged Shutdown (Oregon Health Agency Public Health Division, 2020)	*Legionella*, harmful bacteria, lead, copper	Flush weekly (some considerations given)	NO	NO— But mentioned as preventative	NO	NO	NO	No	Coliform
Arkansas Department of Health Engineering (2020) Flushing Guidance for Buildings with Low Occupancy or No Occupancy During Covid‐19 [33]	Microbial, chemical (lead, copper)	Occasionally flushed	YES—Sediments	YES—Limited instructions	SOME SPEICIFC ACTIONS	NO	NO	NO	Disinfectant Bacteriological
Public Health Madison & Dane County (2020) Water Quality and Your Business: Tips for Re‐opening After Closure Make Sure Your Building's Water System and Devices Are Safe to Use (Public Health Madison & Dane County, 2020)	*Legionella*	Routine flushing	NO	YES—Considerations given including time‐based	NO	NO	YES—Reuse wastewater	NO	NO
City of Durham (NC) (2020) Flushing Water Systems for Reopening (City of Durham, n.d.)	Disease causing microorganisms, corrosion control can be impacted	Preventative flushing	NO	YES—Limited instructions	NO	NO	YES—Capture and reuse water for outdoor use	NO	NO
Minnesota Department of Health (2020) COVID‐19 Reopening Guidance for Noncommunity Public Water Systems (v2) (Minnesota Department of Health, 2020)	*Legionella*, sediment, loss of residual, lead and copper, bacteria in equipment	Flushing as part of reopening plan	YES	YES	YES—Well and storage tanks	NO	NO	NO	Coliforms
Vancouver Coastal Health (2020) Water Stagnation Risks Due to Prolonged Reduced Building Occupancy (Vancouver Coastal Health, 2020)	*Legionella pneumophila*	Flush periodically, maintain hot water, reduce access	YES—Several elements added	NO—But periodic flushing during closure is briefly mentioned	YES—Clean pools	NO	YES—Consult water management plan	NO	NO
Ohio Environmental Protection Agency and Ohio Department of Health (2020) Water Quality Recommendations for Opening Closed or Less Frequently Used Buildings (Ohio Environmental Protection Agency and Ohio Department of Health, 2020)^b^	Metals (lead and copper), opportunistic pathogens (*Legionella*, *Pseudomonas*, nontuberculosis mycobacteria), organics (disinfection byproducts, trihalomethanes and haloacetic acids)	Water management plans, flushing as a preventative measure	NO—But be aware of other hazards	YES—Some considerations given	NO	YES—Use a professional	YES—Continuous disinfection is an option	YES—PPE mentioned	Inorganic, Bacteriological, Unsafe metals, Microbial pathogens, Pressure, Disinfectant residual, Temperature
Nongovernmental organizations									
ESPRI (2020) v1: Coronavirus Building Flushing Guidance [no differences found in version 2] (ESPRI, AH Environmental Consultants, et al., 2020) **Additional (released May 12, 2020) “ “Reducing Risk to Staff Flushing Buildings” (ESPRI, 2020)^a^	Disease causing microorganism, *Legionella pneumophila*, Toxic metals such as lead. Harmful substances such as disinfection byproducts.	Keep water heaters on, Routine flushing, Create water management plan, Shock disinfection can be considered	YES	YES—Detail instructions including time‐based	SEVERAL SPECIFIC ACTIONS	YES—With certain conditions	YES—Ongoing flushing 1/week for 12 weeks	YES—With shock disinfection. **additional document mentions PPE, aerosol reduction techniques	Disinfectant residual
IAPMO (2020) Rehabilitating Stagnant Building Water Systems (Demarco, 2020)	*Legionella* and other pathogens, corrosion issues, off color and taste	Routine flushing (3–4 days)	NO	YES—Some instructions including time‐based	SEVERAL SPECIFIC ACTIONS	YES—Some systems	YES—Other building systems, floor drains, filter maintenance	YES—High levels of pathogens—Open valves slowly	*Legionella* (some buildings)
American Water (2020) Has your facility been closed for weeks? Flush the Pipes (American Water, 2020)	Lead, *Legionella*	Routine flushing (biweekly)	NO	YES—Some instructions including time‐based	NO	NO	NO	NO	NO

a Paper coauthor William Rhoads advised on document; perhaps affecting results.

b COVID‐19 closures were not specifically mentioned, but COVID‐19 is mentioned in an appendix document, and the authors indicated this document was written in response to COVID‐19 closures.

### 3.3 Prevention of water quality issues during periods of low use

Buildings or entire communities can experience long‐term periods of low or no use (e.g., schools over the summer, ski resort/snowbird communities, buildings unoccupied between owners, water shutoffs). The buildings impacted by COVID‐19 stay‐at‐home orders may have had either reduced or no water use for weeks to months. The actions described here may be helpful to implement during stagnation and are recommended in some guidance documents (Table 2).

**Figure 3:**
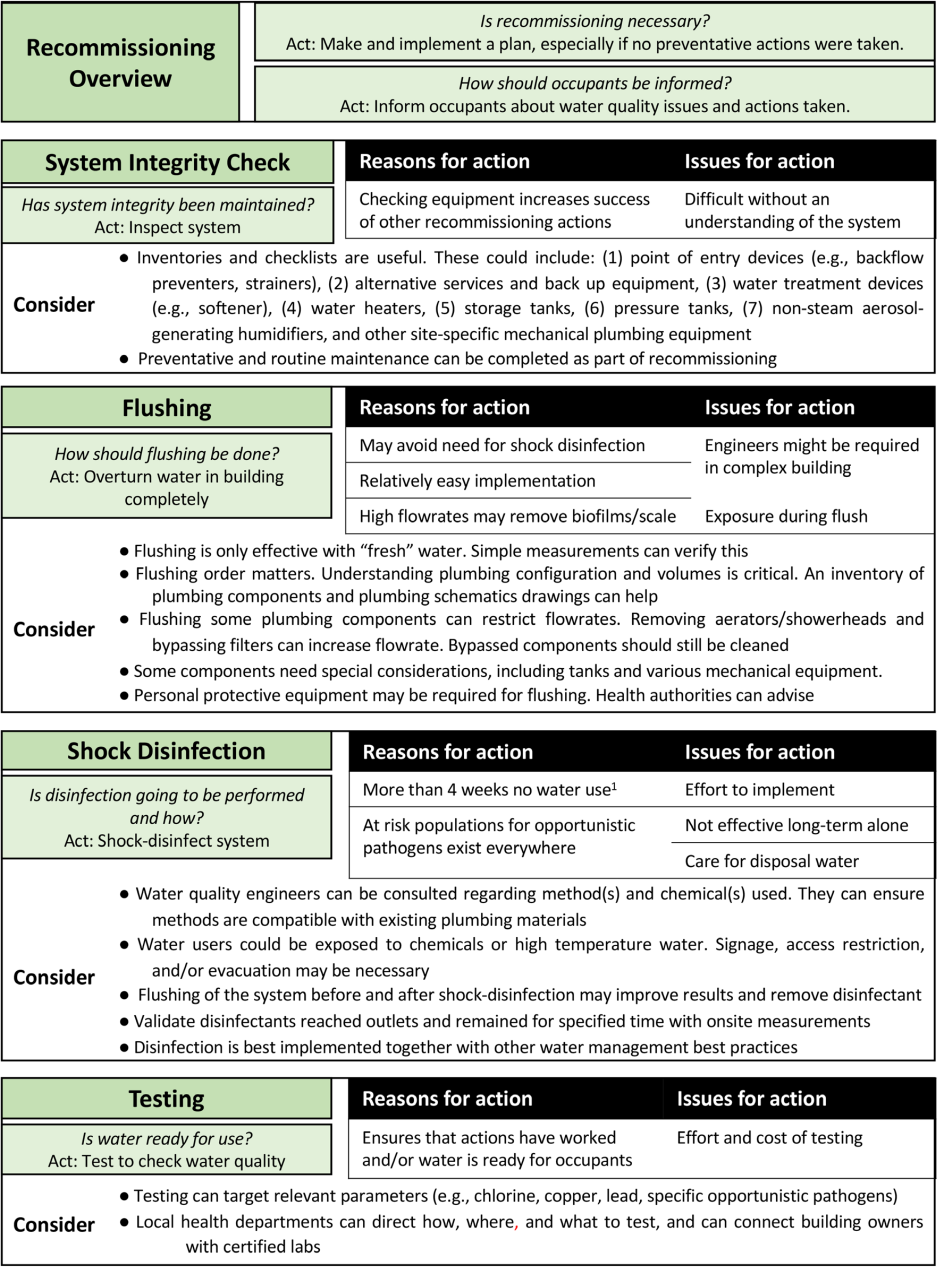
Considerations for recommissioning guidance in six major categories: recommissioning necessity, informing occupants, system integrity, flushing considerations, disinfection considerations, and readiness of water for use. ^1^ASHRAE 188 is an adoptable standard focused on *Legionella* contamination and is the only guidance regarding length of closure that may prompt the recommendation for recommissioning actions; it may not apply to all contaminants discussed; ^2^multifamily residential, >10 stories tall, healthcare facility, patient stays >24 hr, housing or treating immunocompromised individuals, housing >65‐year‐old occupants

Routine flushing introduces freshwater to the system regularly to help prevent problems from developing. It could be used to remediate problems if performed frequently or could offset partial capacity during a ramp‐up of economic activity (Meyers, Luna, & Willon, 2020). BWMPs often contain provisions for weekly flushing of “unused” or “unoccupied” outlets (NASEM, 2019) in otherwise occupied buildings, but criteria for its efficacy have not been thoroughly documented. Necessary frequency is especially difficult to determine. Weekly flushing may be insufficient for effective *Legionella* control due to: (a) plumbing design, hydraulic balancing, or temperature issues; (b) complexity of components such as electronic faucets and thermal mixing valves; and (c) stored volume of water relative to water use (i.e., incomplete turnover). In one hospital with hot water recirculating temperatures that were inadequate to prevent *Legionella* growth (<45°C), a flushing frequency of every 2 hr was required to reduce culturable *Legionella* numbers to “acceptable” levels (Totaro et al., 2018).This flushing frequency is likely only achievable with auto‐flush faucets or solenoid valves and is a clear indication that, for some systems, flushing alone may not achieve acceptable results. Flushing recommendations generally rely upon the assumption that water delivered to the building and used for flushing has a growth deterrent (high temperatures or disinfectant) and a corrosion control component, which may not be the case (Branz et al., 2017; Connexion, 2020; Salehi et al., 2020). Flushing as a preventative measure in completely unoccupied buildings has not been studied previously. Considerations for the development of plans for flushing (i.e., necessary actions, order of actions, duration of flushing) are discussed in Section 3.5 (Figure 3).

Water heater operation can be altered in periods of low or no water use in the building (e.g., turning off a water heater in a summer home during winter). *Legionella* management in large buildings typically relies on thermal control (Bédard et al., 2015, 2016; NASEM, 2019). In large buildings, recirculation loops (Table 1) are often used to move hot water continuously throughout the building, reducing the time for hot water delivery and maintaining high temperatures for stored volumes. For thermal controls to remain effective, both heaters and recirculation lines should maintain high temperatures. However, this approach should be combined with regular flushing of distal pipes at the POU, which can be maintained at ideal opportunistic pathogen growth temperatures during periods of nonuse (Rhoads, Ji, Pruden, & Edwards, 2015). If water heaters or recirculation pumps are completely shut down, this may save energy and allow systems to cool to suboptimal growth ranges. The latter has never been studied but is recommended by some (Table 2). If hot water systems are allowed to cool, adequate amounts of flushing should be performed to maintain a disinfectant residual throughout the hot water system, which may be difficult due to disinfectant reactions with plumbing (e.g., water heater sediments).

Draining building water systems is sometimes carried out when water systems are purposefully shut down for extended periods (e.g., for construction, summer homes in winter). This may prevent growth in water but can introduce many other issues. Plumbing is designed to maintain pressure, and drainage could introduce backflows and contamination from other water systems, such as cooling towers and fire protection systems, if effective backflow prevention is not in place. Refilling systems may result in the destabilization of sediments and biofilms or the introduction of external contaminants to the pipes. Shock disinfection may be necessary at startup after depressurization: If depressurization is thought to have occurred in seasonal potable water systems, additional shock disinfection is recommended (US EPA Region 8, 2020). While one guidance released regarding COVID‐19 building closures advised for draining plumbing (PHE, 2020), another guidance explicitly advises against it due to the pockets of water likely to remain in plumbing (ESGL, 2020). Drained systems likely have different growth conditions, which may induce mold or other organisms to grow. Plumbers are likely needed to safely drain and restart drained plumbing systems, and this procedure may not be feasible for many buildings (e.g., with continued occupancy by essential employees).

Water utility distribution networks suffer similar stagnation issues to buildings. As the efficacy of periodic flushing depends on the water supplied by the water utility, utilities play a role in the prevention of building water quality issues. A disinfectant residual should be present but may be harder to achieve with reduced system demand. Utilities may increase the concentration of disinfectant residual in their distribution system, which has precedent in this and other emergency situations (Branz et al., 2017; Connexion, 2020). This must be weighed against increased disinfection byproduct formation. Utilities may also more closely review routine water quality monitoring data, implement focused flushing efforts (Judd, 2020), or install auto‐flushers to increase delivery of disinfectant residual.

### 3.4 Recovering plumbing after periods of low or nonuse

If no preventative actions are applied, a process called recommissioning may be needed (Figure 3). No consensus was found in literature for the length of time a building can remain unoccupied or have low occupancy before it should be formally recommissioned or for the extent of actions that should be performed. Several actions described in Table 3 are suggested for the annual restart of seasonal potable water systems (e.g., campgrounds, fairgrounds) and as part of initial building commissioning if building occupancy is delayed. Specific care must be taken in adapting these documents as the complexities and variability of large building water systems may not be considered. Moreover, the continued occupancy of some buildings during prolonged stagnation (e.g., essential staff) may not be compatible with certain actions.

**Table 3:** Attributes of actions suggested or required for building startup, commissioning, and recommissioning in referenced documents, codes, and standards

Documents, standards, and codes	Startup/commissioning/recommissioning actions suggested/required				
	Inspection	Flushing and cleaning	Shock disinfection	Safety explicitly mentioned	Testing
UPC (2018): Chapter 6 Water Supply and Distribution (IAPMO, 2018)	Prescriptive actions for each installation step	The system shall be flushed with clean, potable water until potable water appears at the points of the outlets.	The system must be disinfected with specific methods after flushing potable water appears at the points of the outlet.	Not mentioned	Upon completion, the system should be tested with water or air. Test pressures are mentioned.
IPC (2018): Chapter 6 Water Supply and Distribution (International Code Council (ICC), 2018)	Prescriptive actions for each installation step	After construction, the system should be purged of deleterious material	The system must be disinfected with specific methods after flushing potable water appears at the points of the outlet.	Not mentioned	“Bacteriological examination” after disinfection
Revised Total Coliform Rule Checklist (2016) (ADEQ, 2015; US EPA Region 8, 2020; USEPA, 2013)	Inspection of source, storage, and pipes	Flushing stagnant volume required even if system remains pressurized. Flushing recommended after disinfection. Clean/flush out tanks.	Add disinfectant w/ directed dosage, details provided vary across states. Fill system with chlorinated water completely. Let sit 24 hr. Flush. Keep chlorinated water away from septic tanks and surface water.	Not mentioned	Coliform bacteria sample
AWWA 651: Disinfecting Water Mains (AWWA, 2014)	Recommended	Recommended	Required. See Table 2	Concerns for the safety of workers and the public is mentioned.	Coliform bacteria sample
AWWA 652: Disinfecting Water Storage Facilities (AWWA, 1992)	Recommended	Not mentioned. High‐pressure water jet, sweeping, scrubbing, or equally effective means.	Required. See Table 2	Concerns for the safety of workers is mentioned.	Coliform bacteria sample
WHO (2011): Water Safety in Buildings^a^ (WHO, 2011)	Recommended	Recommended for routine use	Adding chlorine compound to the storage tank to have 20–50 mg/L free residual chlorine concentration. Run all taps to smell the chlorine at all fixtures, then close all taps and allow the system to be stagnant at least 1 hr for 50 mg/L and 2 hr 50 mg/L. Then flush the taps to obtain the normal free chlorine level.	Concerns for the safety of building occupants	Free chlorine measurement to make sure super‐chlorinated water if flushed out of the system.

a Additional relevant consideration of WHO (2010) guidance: It is important to keep all finished parts of the water installation dry until the whole system is commissioned for routine operation. If this is not possible, sections that remain stagnant for extended periods should be thoroughly drained and disinfected prior to the system being commissioned; keep all finished parts of the water installation dry; Water‐using devices, end‐of‐plumbing devices, and point of‐use devices should be maintained to minimize microbial growth. These devices should be decommissioned when not in use and, where possible, drained. Water‐using devices will often require decontamination prior to being returned to service; as part of remediation, contaminated drinking water will need to be flushed from the entire distribution system, including water‐using devices, POU and end‐of‐pipe devices. Treatment systems such as water softeners and filtration systems will need to be regenerated, backwashed, or recommissioned before being returned to service. Small filters at POU could harbor contamination and may need replacing. At the time of commissioning, water quality should be documented by hygienic testing of microbial and chemical quality in an adequate set of drinking water samples. Initial higher‐intensity monitoring (additional samples and parameters) might be necessary depending on intended use of the facility, outcomes of inspection, any irregularities during construction or commissioning, and delays in beginning of regular use. In these cases, a water quality expert should be consulted.

System integrity should be checked before taking any remedial actions. For buildings, this could involve the inspection of mechanical and plumbing components (Table 1) to identify leaks, depressurization, adequate backflow prevention, and assessment of functionality (e.g., hot water supply and return temperatures, on‐site disinfectant dosing correctly). The performance of routine maintenance or startup procedures if equipment was taken offline can also be considered.

Recommissioning flushing has similar goals and considerations as routine flushing. There is precedent for using flushing alone to restart water systems that have experienced extended stagnation but maintained pressure (ADEQ, 2015; US EPA Region 8, 2020). However, this flushing‐only strategy is targeted for the reduction of coliforms (Total Coliform Rule (Code of Federal Regulations, 2011)) or lead (flushing for schools (EPA, 2018)), which can be removed relatively easily. Flushing cannot eliminate biofilms where opportunistic pathogens can continue to grow, and it is unclear how flushing alone would impact opportunistic pathogen recurrence. Especially with a slow ramp‐up of building activity (i.e., phase plan for COVID‐19 pandemic recovery (Meyers et al., 2020)), initial flushes might need to be paired with routine flushing to introduce more freshwater to pipes. The development of a flushing plan is discussed in Section 3.5.

Flushing after long stagnation also requires the additional consideration of worker safety. Initial flushes of stagnant water can sometimes release high concentrations of chemical and microbiological contaminants due to high shear stress (Lehtola, Miettinen, Hirvonen, Vartiainen, & Martikainen, 2007) and in situ reactions (SI‐1, reactions occurring during stagnation). To reduce exposure risk, workers responsible for flushing can be screened for preexisting conditions that may make them vulnerable (e.g., to opportunistic pathogens), and/or personal protective equipment (PPE) can be used (Table 2). The Occupational Safety and Health Administration (OSHA) mentions N95 respirators but recommends voluntary use of N100 “if *Legionella* contamination is possible” (OSHA, n.d.). Some COVID‐19 guidance recommends P100 HEPA filter respirators when sampling building water and *Legionella* may be present (AIHA, 2020) as *Legionella* or other pathogen‐containing aerosols can accumulate in the room as flushing is performed. The Centers for Disease Control and Prevention (CDC) recommended, to authors of this study, that local health authorities should be consulted to determine appropriate PPE (CDCInfo, Personal communication, Atlanta, GA, April 1, 2020). The global shortages in critical PPE (e.g., gloves, masks) (Parshley, 2020) surrounding the COVID‐19 pandemic must also be considered. To further reduce exposure, flushing can also be conducted in a manner that reduces water splashing and aerosolization (CDC, n.d.) (e.g., hoses to connect spigots to drains, towels and bags placed over faucets and showerheads, covering toilets). Increasing ventilation can also help to reduce aerosols during flushing activities.

It may be beneficial to coordinate recommissioning flushing with actions taken by the local utility. Customers could coordinate building flushing with utility hydrant flushing efforts or conduct flushing during periods when utilities temporarily boost disinfectant levels. It is possible that many buildings flushing concurrently could impact local buried water distribution system pressure (Johnson, 2014).

Shock disinfection introduces a high concentration of disinfectant or high temperature for a relatively short period of time to reduce the presence of microorganisms in the system. This procedure may reduce biofilms but is not designed to eliminate biofilms. The practice is common for initial building commissioning and remediation of *Legionella* colonization. The American Society of Heating Refrigeration and Air‐conditioning Engineers Standard 188 (ASHRAE 188 (ASHRAE Standards Committee, 2018)) outlines that shock disinfection after construction should occur within 3 weeks of planned occupancy. If occupancy is delayed more than 4 weeks, another shock disinfection is required prior to occupancy. It is unclear how this would apply for buildings that have already been occupied or have continued low occupancy. Another challenge is that the ASHRAE 188 action thresholds (e.g., 3 and 4 weeks) are not based on peer‐reviewed studies with supporting evidence.

Free chlorine, chlorine dioxide, chloramines, and thermal shock have been used successfully for remediating *Legionella* growth (NASEM, 2019; US EPA, 2016). Some COVID‐19 guidance recommends shock disinfection (Table 2) (International Code Council, 2020). Targeted approaches to disinfect plumbing associated with high‐exposure activities for inhalation of aerosols (e.g., showering, Jacuzzis) have been used in the past and are suggested by the CDC in their COVID‐19 building system guidance (CDC, 2020).

The implementation of shock disinfection likely requires the assistance of professionals to ensure efficacy and safety. Disinfection recommendations for commissioning procedures would have to be adapted (Table 4). In short, all parts of the system should be exposed to water with an inhibitory temperature or with disinfectants for recommended durations to achieve sufficient contact time. Flushing is recommended both before and after the procedure to remove loose deposits and high levels of chemical disinfectants. A building water system risk assessment (performed by professionals) can help identify any secondary issues with water system operation (e.g., unbalanced hot water system (Bédard, Boppe, et al., 2016; Bédard, Fey, et al., 2015; Boppe et al., 2016; NASEM, 2019)), thus maximizing procedure efficiency. Material compatibility can be an issue, with shock disinfection causing plumbing leaks and damage (Christensen, 2003; Mead, Lawson, & Patterson, 1988; Raetz, 2010; Rockaway, Wiling, & Schreck, 2007). To avoid dermal and inhalation exposure, it may be necessary to prevent building entrance or post clear signage to warn of harmful chemicals or temperatures. Disposal of water with high chemical concentrations may require pretreatment or coordination with local wastewater authorities. Lower dosage limits combined with increased contact time may be desirable to limit potential issues with high chemical doses (i.e., pipe damage, disposal).

**Table 4:** Comparison of disinfection methods from plumbing codes, AWWA standards for water utility infrastructure, and ASHRAE guideline 12‐2000^a^

Method name	Initial chlorine level/temperature to be maintained	Minimum contact time	Required level after contact
*Uniform Plumbing Code (IAPMO*, *2018* *)*; *International Plumbing Code (International Code Council (ICC)*, 2018 *)*			
Option 1	50 mg/L	24 hr	No level reported
Option 2	200 mg/L	3 hr	No level reported
*AWWA Standard C651‐14*, *Disinfecting Water Mains (*AWWA, 2014 *)* b [46]			
Tablet	25 mg/L	24 hr	0.2 mg/L After 24 hr
Continuous feed	25 mg/L	24 hr	10 mg/L After 24 hr
Slug	100 mg/L	3 hr	Not applicable
Spray	200 mg/L	Not applicable	Not applicable
*AWWA Standard C652‐92*, *Storage Facility Disinfection (*AWWA, *1992* *)*			
Method 1 (full storage)	Achieve 10 mg/L after the appropriate 6 hr or 24 hr period.	6 hr if gaseous chlorine feed used; 24 hr if calcium or sodium hypo used	10 mg/L
Method 2 (spray or painting)	200 mg/L	0.5 hr	Not applicable
Method 3 (full storage)	50 mg/L	24 hr	2 mg/L
*ASHRAE Guideline 12–2000* a (ASHRAE, 2000)			
Chemical shock	To discretion of building owner; indicates that AWWA C651‐14 should not be used (5.5.1)		
Thermal shock	≥70 °C	20 min at all outlets^c^	During flushing

a based on public draft review February 2018.

b These guidelines are not intended for building use.

c Use with caution; thermal capacity of heaters may not be capable of supplying this temperature given flushing demand. Performing shock treatment in phases to allow water heater recovery may decrease efficacy.

Testing, while not typically required for occupied buildings, is the most definitive way to ensure that water in a building is ready for use. Requiring testing after disaster has precedent: When volatile organic compounds were discovered in drinking water after a wildfire in Paradise, California, the local health authority required tests prior to occupancy (BCHD, 2019; Proctor, Lee, Yu, Shah, & Whelton, 2020). Moreover, some North American authorities require testing of lead and copper in schools (Indiana General Assembly, 2020; NY State, 2015). Testing has been recommended in some COVID‐19 guidance documents (Table 2) to validate flushing and recommissioning practices. If conducted, testing should focus on relevant water quality parameters. Testing for disinfectant residual, which is required with the startup of seasonal potable water systems, can easily be performed on site with inexpensive hand‐held instruments. Testing for metals (e.g., lead, copper) can be accomplished using a certified lab. Total coliform or *E. coli* testing, frequently used to certify seasonal systems or buildings for occupancy after construction (ADEQ, 2015; US EPA Region 8, 2020; USEPA, 2013), has low relevance in occupied pressurized buildings. General bacteria testing (e.g., heterotrophic plate count, HPC) may be more relevant to determine the success of shock disinfection, but HPC results are difficult to interpret because normal use results in high and variable counts in buildings (Inkinen et al., 2014; Lautenschlager, Boon, Wang, Egli, & Hammes, 2010; Nguyen et al., 2012; Salehi et al., 2018; Siebel, Wang, Egli, & Hammes, 2016; Zlatanović, van der Hoek, & Vreeburg, 2017). Importantly, HPC levels have not been associated with any known health impacts. Testing for opportunistic pathogens is more relevant for understanding health risks and comparing measured concentrations with concentration limits recommended for reducing risk (Hamilton et al., 2019; Whiley, 2017). However, choosing which one(s) to test for, how/where/when to collect samples (e.g., first flush versus after flushing for several minutes), and how to interpret results requires professional assistance due to the variability in water quality within a building (Bédard et al., 2018; Inkinen et al., 2014; Salehi et al., 2020). Advice for regular *Legionella* sampling in other countries may be a useful start point (NASEM, 2019). Testing should be conducted through a certified lab, and results are not available for 7 days or more. Testing recommendations in guidance documents often lack the necessary specificity, especially regarding where to test (Table 2).

### 3.5 Considerations for flushing plans

Guidance for flushing, whether for routine or recommissioning flushing, must account for variability in building water systems. The development of site‐specific flushing plans is necessary. Estimating the total volume in the water system, or diagnostic testing (disinfectant residual and/or temperature), can help determine how much water to flush at each location. All flushing procedures should begin by establishing freshwater at the POE (i.e., adequate flushing to clear the service line and any stagnant water in the distribution system) and then progressing through the system in a “downstream” fashion, as described below. An inventory of water outlets and devices will help ensure water movement at all taps. Flushing plans may vary slightly if they are conducted periodically versus after extended stagnation; for instance, recommissioning flushing may include draining and cleaning of storage tanks, whereas routine flushing may not. The time, effort, and cost (e.g., water price (Muscarella, 2004)) of flushing will vary considerably among buildings.

**Figure 4:**
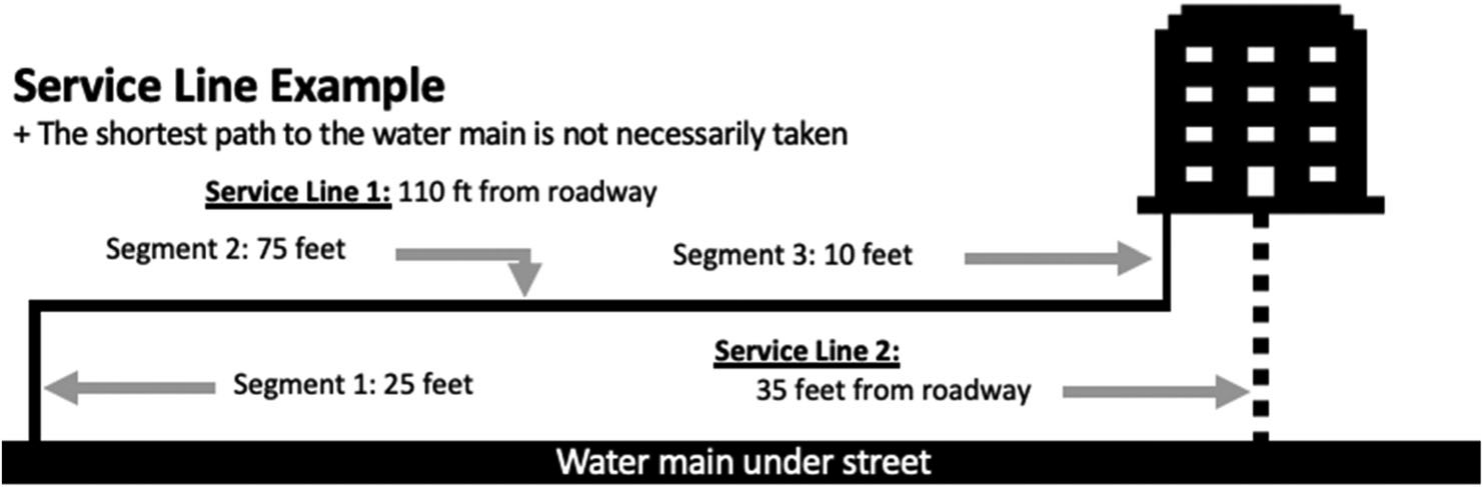
Example variation in the length of an actual service line from water main to an actual building water system

Service lines provide water to the building for cold, hot, and other property water systems (e.g., sprinkler systems (AWWA, 2018), cooling towers). The water volume stored in a commercial building service line can range from tens of gallons to thousands of gallons (Ra, Montagnino, Proctor, & Whelton, 2020) depending on the property design (Figure 4). It is critical that water is flushed at the building POE first to avoid drawing stagnant or potentially contaminated water into plumbing. Multiple POEs may also exist in a building. Conducting POE flushing in conjunction with hydrant flushing—either by the utility or with privately owned fire hydrants (e.g., on campuses)—may be considered to clear service lines (Ra et al., 2020). It is advisable to confirm the presence of disinfectant residual at the POE as distribution system water quality may be degraded.

Mechanical plumbing equipment located in mechanical spaces and “upstream” of the main building piping network (Table 1) must be considered. Bacterial growth, including pathogens, has been associated with this equipment and with subsequent disease cases (Bédard et al., 2016; Borella et al., 2004; Garrison et al., 2016; Stamm, Engelhard, & Parsons, 1969). Volume stored in the devices must be overturned, which can be difficult because of nonideal flow through them (e.g., water heaters; Hawes et al., 2017). As buildings have a wide variety of devices, general guidance should require inventorying devices. Several guidance documents recently released for homes (EGLE, 2020; Ohio Environmental Protection Agency, 2020) and buildings (Table 2) with stagnant/shutoff water fail to account for all devices. In the case of recommissioning, additional action may be needed, including ensuring the equipment is still functioning or draining/cleaning. Manufacturer guidance does not typically cover prolonged stagnation events. Routine maintenance and initial startup procedures (e.g., softener resin replacement or cleaning and disinfection, filter replacement) can serve as a starting point. Adapting existing recommendations should focus on ways to fully overturn the storage volume and remove accumulated sediment/biofilm (Masters et al., 2018; Rhoads et al., 2020).

Plumbing configuration, including pipe length, diameter, and layout, can vary greatly (Figure 2). Site‐specific configurations will affect the water volume (and time) needed to flush each tap, toilet, or device and the order in which outlets should be flushed. Typical nonresidential plumbing systems in large buildings have a trunk‐and‐branch design with one or more risers and headers with branches to individual outlets but may have much more complexity (e.g., multiple pressure zones with tanks in high‐rise buildings, Table 1). Smaller systems may also have a manifold design. Implementing effective protocols may require access to plumbing plans (or as‐built drawings, if available) and/or building personnel knowledge of system design and operation. As‐built construction drawings may also be useful in inventorying every water outlet (e.g., outdoor spigots, forgotten taps) so that every pipe and location is flushed. Dead ends (pipes that lead to nowhere) can also exist in buildings, especially if buildings have been remodeled or had a change in use. Dead‐end pipes will require professional help to flush (e.g., with depressurization); it is best to identify and cap them as close to main branches as possible.

End‐use devices (i.e., appliances, Table 1) also have internal water storage, and many become colonized by pathogens (Beach et al., 2003; Callewaert, Van Nevel, Kerckhof, Granitsiotis, & Boon, 2015; Garvey, Bradley, Tracey, & Oppenheim, 2016; Graman, Quinlan, & Rank, 1997; Ortolano et al., 2005; Sacchetti, De Luca, Guberti, & Zanetti, 2015). Yet, they are absent from some COVID‐19‐inspired building water system flushing guidance (Table 2). These devices' volumes can be overturned by running them or manual action (e.g., discarding several batches of new ice). For recommissioning, routine maintenance of all devices (e.g., replacement of filters) should also be considered. Medical and dental facilities with specific sterile water or special application appliances (e.g., dialysis, heater–cooler machines) must be particularly carefully maintained and cleaned. Procedures and manufacturer protocols for these devices should always be consulted (Allen et al., 2017; Garvey et al., 2016; Muscarella, 2004; ProEdge Dental, 2020; van Ingen et al., 2017).

Fixtures such as faucets, aerators, fountains (bubblers), thermostatic mixing valves, showerheads, and shower hoses can be relatively easily removed, cleaned, and/or discarded. Pathogen growth and heavy metal accumulation (e.g., particles of copper, iron, lead) have been associated with these plumbing components (Bédard et al., 2015; Cohen et al., 2017; Huang & Lin, 2007; Kappstein, Grundmann, Hauer, & Niemeyer, 2000; Proctor, Reimann, Vriens, & Hammes, 2018; Shaw et al., 2018; Sydnor et al., 2012; Takajo et al., 2019; Verweij et al., 1998; Wang, Chen, Lin, Chang, & Chen, 2009; Weber, Rutala, Blanchet, Jordan, & Gergen, 1999; Whiley, Giglio, & Bentham, 2015). Thermostatic mixing valves, used in showers and faucets to mix hot and cold water to prevent scalding, have been identified as particularly problematic for the growth of *Legionella* (Niedeveld, Pet, & Meenhorst, 1986; van der Lugt et al., 2017; Van Hoof, Hornstra, Van Der Blom, Nuijten, & Van Der Wielen, 2014). Cleaning such devices is recommended for normal maintenance (Masters et al., 2018; NASEM, 2019; Health and Safety Executive (HSE), 2013; Castex & Houssin, 2005), and recommissioning may be an opportune time for these practices. At a minimum, these devices should be checked for functionality as the release of sediment during flushing can cause them to leak or become plugged, potentially creating a cross‐connection between hot and cold water systems. While not directly part of water delivery, sink drains can be a source of pathogens, contaminating faucet aerators in hospitals (Parkes & Hota, 2018), and thus, cleaning and disinfection should be considered.

Wastewater generated during flushing must also be considered. If the building utilizes an on‐site septic system, special care must be taken not to overload and flood the system as this can permanently damage the tank and leaching field. Any flushing procedure should ensure that drainage capacity can be met, and flushing must be monitored to avoid flooding (i.e., with drain blockage) and cross‐connections.

Flow rate during flushing is an important consideration, especially as it dictates how long it will take to remove water from plumbing. Some experts suggest low flow rates at taps to minimize aerosolization (Lee, 2020b). Flushing at high flow rates can mobilize loose deposits and biofilm from pipe walls, which may be desirable during recommissioning, but requires several considerations. There is no consensus among experts, and a mixed approach (i.e., starting low and increasing flow rate as water quality improves) could be used.

Mobilizing deposits requires water to move at high velocities. For water mains (4–16 in. diameter [100–400 mm]), a sustained water velocity of 3 ft/s [0.9 m/s] achieved 2.5‐log removal of sand particles (Kirmeyer et al., 2014). For a similar velocity in the smaller pipes of plumbing, very high flow rates would be needed: 2 in. [50 mm] (34 gpm, 129 lpm), 1 1/2 in. [40 mm] (19 gpm, 72 lpm), 1 in. [25 mm] (9 gpm, 34 lpm), and ¾ inch [20 mm] (5 gpm, 19 lpm), ⅝ inch (3 gpm, 11 lpm). Achieving such high flow rates may require additional effort. Removing aerators increased flow rates by 20%–80% (Hawes et al., 2017), but removing these devices can also require special tools or be difficult because of scale buildup. Devices and equipment can reduce water flow rate (e.g., filters, softeners) (Baranovsky et al., 2018), so bypassing devices may be beneficial during downstream flushing, but bypassed devices would still need to be considered in a flushing/cleaning protocol. Guidance documents often recommend opening all faucets at once (i.e., lead service line flushing guidance, designed to maximize flow rate in a service line (Water Works Association, 2017)), but this can be logistically challenging.

High flow rate flushing can also cause issues. To minimize the water hammer effect, a water velocity less than 10 ft/s [3 m/s] is recommended (Angers, 2002). It may also be difficult to maintain pressure during high flow rate flushing, especially in buildings that are large, improperly designed, or that have corrosion issues. If pressure is not maintained, then the resulting reduced flow (velocity) in individual distal pipes could be ineffective for flushing or result in the deposition of particles (i.e., lead) that were dislodged from trunks or service lines. Depressurization, which could trigger a need for disinfection, and back‐siphonage, the reversal of flow direction (Hawes et al., 2017), can also occur. To avoid sediment deposition of service line sediments (e.g., lead), the service line can be flushed first at the POE. Opening only a subset of fixtures (i.e., by pressure zone) may also ease pressure demands.

Diagnostic testing can check that that flushing is complete—that all stagnant water is removed from the system and growth deterrent is delivered to all taps. Diagnostic tests, including turbidity, pH, temperature, specific conductance, and disinfectant residual, are suggested by the US Environmental Protection Agency (USEPA) to determine where water is originating from during flushing (EPA Region 4, 2019). Temperature stabilization (<0.1 °C or <0.2 °F change (EPA Region 4, 2019)) is easy and may indicate that water is coming from the distribution network, but local climate considerations should be taken into account (i.e., water will not get cold in hot climates in summer). Inexpensive hand‐held disinfectant residual monitors can also be used. Disinfectant residual test strips should be used with caution. Care should be taken to test for the correct disinfectant (i.e., free and total chlorine for systems using chlorine versus monochloramine and total chlorine for systems using chloramines; total chlorine alone can be a useful indictor for either system).

A disinfectant residual is unlikely to persist through water heaters, so temperature is used as a flushing diagnostic in hot water systems. This is more difficult than in cold systems but has been successful (Bédard, Fey, et al., 2015; Boppe et al., 2016). A high stable temperature (i.e., 55 °C, recirculating temperature) may be reached for several minutes without drawing significantly from the water heater (i.e., 60 °C, heater set point). If the system is not properly balanced, steady‐state temperatures can vary substantially throughout the building. Changes in building heating and cooling or hot water system operation during shutdown may also affect flushing temperature profiles. Installation of temperature probes may enable building managers to better understand their building water during flushing and normal use.

Flushing duration is extremely difficult to generalize in flushing plans. Widely issued time‐based flushing protocols will be ineffective for some buildings becase of variability in building water systems. For example, using some prescribed times recommended in COVID‐19 building water guidance documents (Table 2) would not suffice for removing the “dead volume” from an out patient healthcare facility, green office building, or a school in which the authors have worked (Montagnino, Ra, Proctor, & Whelton, 2020; Ra et al., 2020; Rhoads et al., 2016). Because of nonideal and nonplug flows in pipes and appliances, replenishing volume will require flushing more volume than is present in the system (Hawes et al., 2017). Even under normal scenarios, residuals can be difficult to achieve at the POU, lengthening the necessary flushing time. For example, >80 minutes of flushing was needed to obtain a residual at distal outlets in one green outpatient healthcare building (Rhoads et al., 2016). Flow rates can vary considerably by tap (i.e., attributable to flow obstruction by scale or fittings), temperature, or time (i.e., inconsistent pressure delivery) within a building. If relying on volume calculations alone, these variations may impact time needed to flush.

### 3.6 Communication

No regulations were found that required building owners to notify building occupants about building water quality. However, a proactive approach to addressing and communicating water quality issues in buildings is generally recommended at both the utility and facility levels (AWWA, 2020). If a communication program is pursued, several items should be considered. Communications about building water health risk should be coordinated with local public health authorities. Example communication messages for building owners, public health authorities, and water utilities related to building water can be found in the supplementary information section (see SI‐2).

Information developed for utilities may be informative for developing materials for building owners to communicate with their occupants. Available materials are focused on utilities communicating with building owners regarding the presence and detection of *Legionella*, lead, disinfection byproducts, and total coliforms (American Water Works Association, n.d.; ASHRAE Standards Committee, 2018; AWWA, 2019; CDC, 2017b, 2017c; CDC et al., 2016; EPA, 2013; Masters et al., 2018; USEPA, 2018a, 2018b). Guidance on utility‐issued boil‐water notices and do‐not‐drink and do‐not‐use notices in escalating order of severity is also available (CDC et al., 2016), but building owners should be aware that these warnings are focused on meeting primary drinking water regulations and are not necessarily protective of public health with respect to opportunistic pathogens.

Public health communications should inform building occupants about building‐specific hazards and preventative or mitigating actions being taken and the reasons for performing those actions. These communications should follow standard approaches of: (a) being tailored to individual building situations; (b) addressing specific occupant concerns; (c) identifying particular risk factors for those potentially exposed (e.g., elderly or immune‐compromised), so individuals can make informed decisions limiting their risk; (d) providing accessible delivery to all building occupants (i.e., sixth to seventh grade reading level, multiple languages, and delivery modes such as email or signage); and (e) communicating specific preventative actions (e.g., not entering the building during flushing periods). Communication regarding risks to *Legionella* exposure has been developed (CDC, 2017a) but has not been tailored for the COVID‐19 situation.

Temporary water use restrictions or guidelines targeting specific actions have been enacted in past disasters. A similar approach may be relevant as COVID‐19 stay‐at‐home orders are lifted to minimize public health impacts from stagnant water. For example, when volatile organic compounds were discovered in drinking water after a wildfire, water use restrictions targeted exposures to volatiles (PID, 2018; Proctor et al., 2020). After extended stagnation, a temporary restriction on showers and other aerosol‐producing devices in affected buildings could be considered. Water use could also be limited to toilet flushing and handwashing. This could help protect a variety of individuals who are at higher risk of opportunistic pathogen infection, including critically ill or highly immunocompromised individuals (neonates, chronic obstructive pulmonary disease [COPD] or chronic lung disease patients, cancer patients), as well as a large fraction of the general population (>60 years old, smokers, diabetics) and, potentially, persons who are recovering from COVID‐19. Vulnerable populations can exist in all buildings and are not always easily identified. One large building owner who contacted the authors has been posting “drinking water out of service signs” at faucets where no residual can be found, which also reduces touch points for COVID‐19 transmission. In extreme contamination situations, building owners could perform a “lockout and tagout” of the affected water fixture or building area (Sonoma State University, 2016).

## 4 CONCLUSIONS

This review was conducted to inform the development of guidance to address water quality concerns in fully or partially shutdown buildings and the reopening/repurposing of other buildings. Reduced or no water use in buildings may present both chemical (lead, copper) and microbiological (opportunistic pathogens) health risks. However, the unprecedented nature of widespread, long‐term building closures has never been studied, and health risks have not been quantified with respect to specific plumbing designs, plumbing features, or operational parameters. Building water quality is the responsibility of the building owner, although codes require that the local health authority (generally referred to in the UPC and IPC) (IAPMO, 2018; International Code Council (ICC), 2018) make decisions about building water system commissioning, and similar responsibility may be conferred for recommissioning. The delivery of high‐quality water requires the cooperation of several diverse stakeholders.

Several efforts were recognized as requiring future investigation, which include the need to

- Evaluate the effectiveness of specific recommissioning actions or series of actions in reducing health risks across plumbing configurations. Documenting success or failure of guidance will allow for improved guidance that minimizes risks and costs.
- Develop methods for determining the frequency, number, and location of representative water samples for a building and the necessary chemical and microbiological analyses needed to adequately assess health risks and inform remedial actions.
- Investigate the factors that control chemical and microbiological water quality characteristics under prolonged stagnation (i.e., months) and strategies to prevent water quality deterioration.

The COVID‐19 response provides an opportunity for health officials, building owners, and utilities to proactively reduce building water system health risks. Coordination of efforts between these entities will enhance success. Evidence‐based standards and guidance are lacking and are needed to address routine building water system maintenance (e.g., flushing), monitoring, and recommissioning procedures (Singh et al., 2020). In the absence of those standards, information contained in this review can help inform and guide health authorities and building officials make building water system and public health decisions. In writing guidance for buildings impacted by the COVID‐19 pandemic, several key facts should be considered:

- Guidance must allow for site‐specific variation in buildings and allow for tailored plans and actions.
- Stagnation duration and severity will vary (e.g., by length of stay‐at‐home order, type of business, and plan for building reoccupancy), and actions may need to be tailored. More complex building plumbing, particularly for higher‐risk occupants, may require more intensive preventative or remedial measures.
- Multiple resources have been developed regarding COVID‐19, but not all are equally reliable or relatable to low occupancy of building closures prompted by the pandemic.
- Clear communication with building occupants and workers performing water system maintenance and recommissioning actions can establish trust and better protect public health.
- With a slow ramp‐up of building occupancy, water stagnation will continue. Repeated actions (e.g., routine flushing) may be necessary to prevent water quality degradation and plumbing damage.
- Emergency preparedness requires forethought. If preventative actions (i.e., routine flushing guidelines) are developed now, they can be implemented now and in response to future disasters that prompt low occupancy or building shutdowns.

## CONFLICTS OF INTEREST

The authors declare no competing interest.

## Supporting information


**Appendix S1.** Supporting Information

## References

[aws21186-bib-0001] ADEQ . (2015). *Revised total coliform rule seasonal start up procedures certification form*. Retrieved from https://legacy.azdeq.gov/environ/water/dw/download/rtcr_seasonal_startup_cert.pdf

[aws21186-bib-0002] AIHA . (2020). *Recovering from COVID‐19 building closures guidance document*. Retrieved from https://www.epa.gov/pesticide-registration/list-n-disinfectants-use-against-sars-cov-2

[aws21186-bib-0003] Allen, K. B. , Yuh, D. D. , Schwartz, S. B. , Lange, R. A. , Hopkins, R. , Bauer, K. , … Wentz, C. (2017). Nontuberculous mycobacterium infections associated with heater‐cooler devices. Annals of Thoracic Surgery, 104, 1237–1242. 10.1016/j.athoracsur.2017.04.067 28821331

[aws21186-bib-0004] American Water . (2020). *Has your facility been closed for weeks ? Flush the pipes*. 53.

[aws21186-bib-0005] American Water Works Association . (2002). *Effects of water age on distribution system water quality background and disclaimer*. Retrieved from http://www.epa.gov/safewater/disinfection/tcr/regulation_revisions.html

[aws21186-bib-0006] American Water Works Association (n.d.). *Sample utility communications plan*. Retrieved from https://www.awwa.org/Policy‐Advocacy/Communications‐Outreach/Public‐Communications‐Toolkit/Sample‐Utility‐Communications‐Plan

[aws21186-bib-0007] American Water Works Association and Association of Metropolitan Water Utilities . (2020). The financial impact of the COVID‐19 crisis on U.S. Drinking Water Utilities.

[aws21186-bib-0008] Angers, J. (2002). Why should we avoid dead ends? Opflow, 28, 10–11. 10.1002/j.1551-8701.2002.tb01678.x

[aws21186-bib-0009] Arkansas Department of Health Engineering . (2020). *Flushing guidance for buildings with low occupancy or no occupancy during Covid‐19*.

[aws21186-bib-0010] Arnold, R. B. , & Edwards, M. (2012). Potential reversal and the effects of flow pattern on galvanic corrosion of Lead. Environmental Science and Technology, 46, 10941–10947. 10.1021/es3017396 22900550

[aws21186-bib-0011] ASHRAE . (2000) *Guideline 12–2000—Minimizing the risk of legionellosis associated with building water systems*. Retrieved from https://www.techstreet.com/ashrae/standards/guideline‐12‐2000‐minimizing‐the‐risk‐of‐legionellosis‐associated‐with‐building‐water‐systems?product_id=232891

[aws21186-bib-0012] ASHRAE Standards Committee . (2018) ANSI/ASHRAE standard 188–2018. Legionellosis: Risk management for building water systems. Atlanta, GA. Retrieved from www.ashrae.org/technology

[aws21186-bib-0013] AWWA . (1992). American Water Works Association ANSI/AWWA C652‐92 AWWA standard for disinfection of water‐storage facilities.

[aws21186-bib-0014] AWWA . (2009). Manual of water supply practices, M36 (3rd Ed.), AWWA water audits and loss control programs). Denver, CO: Author.

[aws21186-bib-0015] AWWA . (2014). ANSI/AWWA C651‐14. AWWA standard disinfecting water mains, Denver, Colorado: AWWA. 10.12999/AWWA.C651.14

[aws21186-bib-0016] AWWA . (2018) Residential fire sprinkler systems guidance for water utilities, Denver, Colorado: AWWA. https://www.awwa.org/Portals/0/AWWA/ETS/Resources/ResidentialFireSprinklerSystems.pdf.

[aws21186-bib-0017] AWWA . (2019) Trending in an Instant. A risk communication guide for water utilities, Denver, Colorado: AWWA.

[aws21186-bib-0018] AWWA . (2020). *Sample utility communications plan*. Retrieved from https://www.awwa.org/Policy‐Advocacy/Communications‐Outreach/Public‐Communications‐Toolkit/Sample‐Utility‐Communications‐Plan

[aws21186-bib-0019] Baranovsky, S. , Jumas‐Bilak, E. , Lotthé, A. , Marchandin, H. , Parer, S. , Hicheri, Y. , & Romano‐Bertrand, S. (2018). Tracking the spread routes of opportunistic premise plumbing pathogens in a haematology unit with water points‐of‐use protected by antimicrobial filters. Journal of Hospital Infection, 98, 53–59. 10.1016/j.jhin.2017.07.028 28760634

[aws21186-bib-0020] BCHD . (2019). *Environmental health issues guidelines for business planning to open in the camp fire affected areas—butte county recovers*. Retrieved from https://buttecountyrecovers.org/environmental-health-issues-guidelines-for-business-planning-to-open-in-the-camp-fire-affected-areas/

[aws21186-bib-0021] Beach, M. , Visvesvara, G., Kolman, J., Waldbillig, T., Weisbuch, J., Amann, J., … Santana, S. (2003, October 11). *Naegleria fowleri in a drinking water system: Two fatal cases of primary amebic meningoencephalitis*, *Arizona*, *2002*. IDSA.

[aws21186-bib-0022] Bédard, E. , Boppe, I. , Kouamé, S. , Martin, P. , Pinsonneault, L. , Valiquette, L. , … Prévost, M. (2016). Combination of heat shock and enhanced thermal regime to control the growth of a persistent legionella pneumophila strain. Pathogens, 5(2), 35. 10.3390/pathogens5020035 PMC4931386 27092528

[aws21186-bib-0023] Bédard, E. , Fey, S. , Charron, D. , Lalancette, C. , Cantin, P. , Dolcé, P. , … Prévost, M. (2015). Temperature diagnostic to identify high risk areas and optimize legionella Pneumophila surveillance in hot water distribution systems. Water Research, 71, 244–256. 10.1016/j.watres.2015.01.006 25622002

[aws21186-bib-0024] Bédard, E. , Laferrière, C. , Charron, D. , Lalancette, C. , Renaud, C. , Desmarais, N. , … Prévost, M. (2015). Post‐outbreak investigation of *Pseudomonas aeruginosa* faucet contamination by quantitative polymerase chain reaction and environmental factors affecting positivity. Infection Control and Hospital Epidemiology, 36, 1337–1343. 10.1017/ice.2015.168 26190556

[aws21186-bib-0025] Bédard, E. , Laferrière, C. , Déziel, E. , & Prévost, M. (2018). Impact of stagnation and sampling volume on water microbial quality monitoring in large buildings. PLoS One, 13, e0199429. 10.1371/journal.pone.0199429 29928013 PMC6013212

[aws21186-bib-0026] Bédard, E. , Lévesque, S. , Martin, P. , Pinsonneault, L. , Paranjape, K. , Lalancette, C. , … Prévost, M. (2016). Energy conservation and the promotion of legionella pneumophila growth: The probable role of heat exchangers in a nosocomial outbreak. Infection Control and Hospital Epidemiology, 37, 1475–1480. 10.1017/ice.2016.205 27640674 PMC5197645

[aws21186-bib-0027] Boppe, I. , Bédard, E. , Taillandier, C. , Lecellier, D. , Nantel‐Gauvin, M. A. , Villion, M. , … Prévost, M. (2016). Investigative approach to improve hot water system hydraulics through temperature monitoring to reduce building environmental quality Hazard associated to legionella. Building and Environment, 108, 230–239. 10.1016/j.buildenv.2016.08.038

[aws21186-bib-0028] Borella, P. , Montagna, M. T. , Romano‐Spica, V. , Stampi, S. , Stancanelli, G. , Triassi, M. , … D'Alcalà, G. R. (2004). Legionella infection risk from domestic hot water. Emerging Infectious Diseases, 10(3), 457–464. 10.3201/eid1003.020707 15109413 PMC3322798

[aws21186-bib-0029] Brandt, M. , Clement, J. , Powell, J. , Casey, R. , Holt, D. , Harris, N. , & Tuan Ta, C. (2005) *Managing distribution retention time to improve water quality‐phase I (AwwaRF report 91006F)*. Denver, CO. Retrieved from https://www.iwapublishing.com/books/9781843399018/managing-distribution-retention-time-improve-water-quality

[aws21186-bib-0030] Branz, A. , Levine, M. , Lehmann, L. , Bastable, A. , Ali, S. I. , Kadir, K. , … Lantagne, D. (2017). Chlorination of drinking water in emergencies: A review of knowledge to develop recommendations for implementation and research needed. Waterlines, 36, 4–39. 10.3362/1756-3488.2017.002

[aws21186-bib-0031] Callewaert, C. , Van Nevel, S. , Kerckhof, F.‐M. , Granitsiotis, M. S. , & Boon, N. (2015). Bacterial exchange in household washing machines. Frontiers in Microbiology, 6, 1381. 10.3389/fmicb.2015.01381 26696989 PMC4672060

[aws21186-bib-0032] Castex, J. , & Houssin, D. . (2005). *L'eau Dans Les E'tablissements de Sante'rance*. Ministère de la Sante' et des Solidarite's.

[aws21186-bib-0033] CBECS 2012 . (2015). *A Look at the U.S. Commercial Building Stock: Results from EIA's 2012 Commercial Buildings Energy Consumption Survey (CBECS). CBECS 2012: Building Stock Results*. Retrieved from https://www.eia.gov/consumption/commercial/reports/2012/buildstock/.

[aws21186-bib-0034] CDC . (2017a). *Developing a water management program to reduce legionella growth and spread in buildings. A practical guide to implementing industry standards*. Retrieved from www.cdc.gov/legionella

[aws21186-bib-0035] CDC . (2017b) *Developing a water management program to reduce legionella growth & spread in buildings: a practical guide to implementing industry standards*. Centers for Disease Control and Prevention. Retrieved from www.cdc.gov/legionella, https://www.cdc.gov/legionella/wmp/toolkit/index.html

[aws21186-bib-0036] CDC . (2017c). *Developing a water management program to Reduce Legionella Growth & Spread in buildings a practical guide to implementing industry standards*. Retrieved from www.cdc.gov/legionella

[aws21186-bib-0037] CDC . (2020, April 3). Guidance for building water systems. CDC. Retrieved from https://www.cdc.gov/coronavirus/2019-ncov/php/building-water-system.html

[aws21186-bib-0038] CDC . *Reduce risk from water*. CDC. Retrieved from https://www.cdc.gov/hai/prevent/environment/water.html

[aws21186-bib-0039] CDC , USEPA , & AWWA . (2016). *Drinking water advisory communication toolbox—2016*. Retrieved from http://www.cdc.gov/healthywater/emergency/dwa-comm-toolbox/index.html

[aws21186-bib-0040] Christensen . (2003). An overview of oxcide: The definitive solution to disinfection in facility water distribution systems & equipment. February 2003.

[aws21186-bib-0041] City of Durham . (n.d.). *Flushing water systems for reopening*. Durham, NC. Retrieved from https://durhamnc.gov/4046/Important-Information-for-Businesses-As

[aws21186-bib-0042] CMS . (2017). *SUBJ: Requirement to reduce legionella risk in healthcare facility water systems to prevent cases and outbreaks of legionnaires' disease (LD)*. Retrived from www.ashrae.org

[aws21186-bib-0043] Code of Federal Regulations (2011). 40 CFR 261.24—Toxicity characteristic.

[aws21186-bib-0044] Cohen, R. , Babushkin, F. , Shimoni, Z. , Cohen, S. , Litig, E. , Shapiro, M. , … Paikin, S. (2017). Water faucets as a source of *Pseudomonas aeruginosa* infection and colonization in neonatal and adult intensive care unit patients. American Journal of Infection Control, 45, 206–209. 10.1016/j.ajic.2016.05.029 27566870

[aws21186-bib-0045] Connecticut Department of Public Health . (2020) Building water system return to service guidance. Hartford, Connecticut: Conneticut Department of Health.

[aws21186-bib-0046] Connexion . (2020, April 5). Extra chlorine in tap water in france due to Covid‐19. *The Connexion*. Retrieved from https://www.connexionfrance.com/French‐news/Extra‐chlorine‐added‐to‐tap‐water‐in‐France‐due‐to‐stagnant‐water‐and‐Covid‐19‐confinement

[aws21186-bib-0047] Demarco, P. (2020). *Rehabilitating stagnant building water systems—A timely reminder from the IAPMO group*. Retrieved from www.iapmo.org

[aws21186-bib-0048] Dias, V. C. F. , Besner, M. C. , & Prévost, M. (2017). Predicting water quality impact after district metered area implementation in a full‐scale drinking water distribution system. Journal—American Water Works Association, 109, E363–E380. 10.5942/jawwa.2017.109.0099

[aws21186-bib-0049] EGLE . (2020). *Flushing your house plumbing system when water services are restored*. Retrieved from www.Michigan.gov/EGLE

[aws21186-bib-0050] Elfland, C. , Paolo, S. , & Marc, E. (2010). Lead‐contaminated water from Brass plumbing devices in new buildings. Journal—American Water Works Association, 102, 66–76. 10.1002/j.1551-8833.2010.tb11340.x

[aws21186-bib-0051] EPA . (2013). *Water security initiative: Interim guidance on developing risk communication plans for drinking water utilities*. www.epa.gov/watersecurity

[aws21186-bib-0052] EPA . (2018, October). *3Ts Flushing best practices*. Office of Ground Water and Drinking Water. EPA 815‐F‐18‐027. Retrieved from https://www.epa.gov/sites/production/files/2018‐09/documents/flushing_best_practices_factsheet_508.pdf

[aws21186-bib-0053] EPA Region 4 . (2019). *Potable water supply sampling*.

[aws21186-bib-0054] ESGL . (2020). *European Society of Clinical Microbiology and Infectious Disease Study Group for Legionella Infections. ESGLI Guidance for Managing Legionella in Building Water Systems during the COVID‐19 Pandemic*. Retrieved from https://www.pwtag.org/guidance-on-temporary-pool-closure/

[aws21186-bib-0055] ESPRI . (2020). *Reducing risk to staff flushing buildings*. Retrieved from www.esprinstitute.org

[aws21186-bib-0056] ESPRI, AH Environmental Consultants , Bartrand, T. , Masters, S. , Hargy, T. , Mccuin, R. , Clancy, J. , Theiss, R. , Pommerenk, P. , Mcnamara, S. , & Hiltebrand, D. (2020) *Building water quality and coronavirus: Flushing guidance for periods of low or no use*. Retrieved from Esprinstitute.Org.

[aws21186-bib-0057] Food & Water Watch . (2018). *America's secret water crisis: National shutoff survey reveals water affordability emergency affecting millions*.

[aws21186-bib-0058] Garrison, L. E. , Kunz, J. M. , Cooley, L. A. , Moore, M. R. , Lucas, C. , Schrag, S. , … Whitney, C. G. (2016). Vital signs: Deficiencies in environmental control identified in outbreaks of legionnaires' disease‐North America, 2000‐2014. Morbidity and Mortality Weekly Report, 65, 576–584. 10.15585/mmwr.mm6522e1 27281485

[aws21186-bib-0059] Garvey, M. I. , Bradley, C. W. , Tracey, J. , & Oppenheim, B. (2016). Continued transmission of *Pseudomonas aeruginosa* from a wash Hand Basin tap in a critical care unit. Journal of Hospital Infection, 94, 8–12. 10.1016/j.jhin.2016.05.004 27249962

[aws21186-bib-0060] Graman, P. S. , Quinlan, G. A. , & Rank, J. A. (1997). Nosocomial Legionellosis traced to a contaminated ice machine. Infection Control and Hospital Epidemiology, 18, 637–640. 10.2307/30141491 9309436

[aws21186-bib-0061] Gupta, L. C. , & Thawari, S. (2016). Plumbing system in high rise building. IJIRST‐International Journal for Innovative Research in Science & Technology, 2, 719–723. www.ijirst.org

[aws21186-bib-0062] Hamilton, K. A. , Hamilton, M. T. , Johnson, W. , Jjemba, P. , Bukhari, Z. , Lechevallier, M. , … Gurian, P. L. (2019). Risk‐based critical concentrations of legionella Pneumophila for indoor residential water uses. Environmental Science and Technology, 53, 4528–4541. 10.1021/acs.est.8b03000 30629886

[aws21186-bib-0063] Hasit, Y. J. , Anderson, J. L. , Parolari, A. J. , Rockaway, T. D. , & French, M. L. (2006). Distribution water quality issues related to new development or low usage. Denver, CO: AWWA Research Foundation.

[aws21186-bib-0064] Hawes, J. K. , Conkling, E. A. , Casteloes, K. S. , Brazeau, R. H. , Salehi, M. , & Whelton, A. J. (2017). Predicting contaminated water removal from residential water heaters under various Flushing scenarios. Journal—American Water Works Association, 109, E332–E352. 10.5942/jawwa.2017.109.0085

[aws21186-bib-0065] Health and Safety Executive (HSE) . (2013). Legionnaires' disease: Technical guidance. Part 2: The control of legionella bacteria in hot and cold water systems, Norwich, England: HSE Books, United Kingdom.

[aws21186-bib-0066] Huang, W.‐K. , & Lin, Y. E. (2007). A controlled study of legionella concentrations in water from faucets with aerators or laminar water flow devices. Infection Control & Hospital Epidemiology, 28, 765–766. 10.1086/516797 17520562

[aws21186-bib-0067] IAPMO . (2018). Uniformed plumbing code (UPC), 28th Edition. Ontario, CA: International Association of Plumbing and Mechanical Officials.

[aws21186-bib-0068] Indiana Department of Environmental Management . (2020). *IDEM guidance document guidance for flushing water systems*. Retrieved from https://engineering.purdue.edu/PlumbingSafety/project

[aws21186-bib-0069] Indiana General Assembly . (2020). House bill 1265—drinking water testing—Indiana general Assembly, 2020 session. Indianapolis, IN: Indiana General Assembly Retrieved from http://iga.in.gov/legislative/2020/bills/house/1265

[aws21186-bib-0070] Indiana State Department of Health . (2020). *Building water system startup guidance*. Retrieved from https://engineering.purdue.edu/PlumbingSafety/covid19/Guidance-Evaluation-Tool.pdf

[aws21186-bib-0071] Inkinen, J. , Kaunisto, T. , Pursiainen, A. , Miettinen, I. T. , Kusnetsov, J. , Riihinen, K. , & Keinänen‐Toivola, M. M. (2014). Drinking water quality and formation of biofilms in an office building during its first year of operation, a full scale study. Water Research, 49, 83–91. 10.1016/j.watres.2013.11.013 24317021

[aws21186-bib-0072] International Code Council . (2020). Guidance for the disinfection of building water systems using the international plumbing code® . Retrieved from www.iccsafe.org

[aws21186-bib-0073] International Code Council (ICC) . (2018). *International plumbing code*. Washington, DC: Author.

[aws21186-bib-0074] Ireland HSA . (2020). Ireland health and safety authority. Control of legionella bacteria during and after the COVID‐19 pandemic.

[aws21186-bib-0075] Jiang, I. (2020, March 24). What is a nonessential business, Essential business during coronavirus? *Business Insider*. Retrieved from https://www.businessinsider.com/what-is-a-nonessential-business-essential-business-coronavirus-2020-3.

[aws21186-bib-0076] Johnson, S. (2014, January 13). WV MetroNews initial do‐not‐use water orders lifted. Flushing Process Begins. *WV MetroNews*. Retrieved from http://wvmetronews.com/2014/01/13/first-do-not-use-water-order-lifted-flushing-process-begins/

[aws21186-bib-0077] Judd, J. (2020, April 25). Reopening businesses reminded to flush water system. *KNSI Radio*. Retrieved from https://knsiradio.com/news/local-news/reopening-businesses-reminded-flush-water-system

[aws21186-bib-0078] Julien, R. , Dreelin, E. , Whelton, A. J. , Lee, J. , Aw, T. G. , Dean, K. , & Mitchell, J. (2020). Knowledge gaps and risks associated with premise plumbing drinking water quality. AWWA Water Science, 2(3), e1177. 10.1002/aws2.1177.

[aws21186-bib-0079] Kappstein, I. , Grundmann, H. , Hauer, T. , & Niemeyer, C. (2000). Aerators as a reservoir of *Acinetobacter junii*: An outbreak of bacteraemia in paediatric oncology patients. Journal of Hospital Infection, 44, 27–30. 10.1053/jhin.1999.0648 10633050

[aws21186-bib-0080] Kirmeyer, G. J. , Thomure, T. M. , Rahman, R. , Marie, J. L. , LeChevallier, M. W. , Yang, J. , … Schneider, O. (2014). Effective microbial control strategies for main breaks and depressurization. Denver, CO: Water Research Foundation Retrieved from http://wioa.org.au/operator_resources/documents/WaterRF2014_Report.pdf

[aws21186-bib-0081] Kurth, J. (2019, August 19). Detroit shut off water to 11,800 homes this year. most are still off. *Bridge Magazine*. Retrieved from https://www.bridgemi.com/urban-affairs/detroit-shut-water-11800-homes-year-most-are-still.

[aws21186-bib-0082] Lautenschlager, K. , Boon, N. , Wang, Y. , Egli, T. , & Hammes, F. (2010). Overnight stagnation of drinking water in household taps induces microbial growth and changes in community composition. Water Research, 44, 4868–4877. 10.1016/j.watres.2010.07.032 20696451

[aws21186-bib-0083] Lee, A. (2020a, March 30). Stay‐at‐home orders: Which states are implementing them. *CNN*. Retrieved from https://www.cnn.com/2020/03/23/us/coronavirus‐which‐states‐stay‐at‐home‐order‐trnd/index.html.

[aws21186-bib-0084] Susanne Lee . (2020b, April 23). How to ensure your building water system is safe during and post COVID‐19. *Webinar*. Retrieved from https://www.rsph.org.uk/events/webinars/how‐to‐ensure‐your‐building‐water‐system‐is‐safe‐during‐and‐post‐covid‐19.html

[aws21186-bib-0085] Lehtola, M. J. , Miettinen, I. T. , Hirvonen, A. , Vartiainen, T. , & Martikainen, P.J. (2007). Effects of water flow regime on water quality in copper and plastic pipes. In *Proceedings of Clima 2007 WellBeing Indoors*.

[aws21186-bib-0086] Lipphaus, P. , Hammes, F. , Kötzsch, S. , Green, J. , Gillespie, S. , & Nocker, A. (2014). Microbiological tap water profile of a medium‐sized building and effect of water stagnation. Environmental Technology, 35, 620–628. 10.1080/09593330.2013.839748 24645441

[aws21186-bib-0087] Lytle, D. A. , & Liggett, J. (2016). Impact of water quality on chlorine demand of corroding copper. Water Research, 92, 11–21. 10.1016/j.watres.2016.01.032 26826646

[aws21186-bib-0088] Masters, S. , Clancy, J.L. , Villegas, S. , LeChevallier, M. , & Bukhari, Z. (2018). *Customer messaging on opportunistic pathogens in plumbing systems* | The Water Research Foundation. Water Research Foundation Project #4664. Retrieved from https://www.waterrf.org/research/projects/customer-messaging-opportunistic-pathogens-plumbing-systems

[aws21186-bib-0089] Mead, P. B. , Lawson, J. M. , & Patterson, J. W. (1988). Chlorination of water supplies to control legionella may corrode the pipes. JAMA, 260, 2216. 10.1001/jama.1988.03410150064019 3172399

[aws21186-bib-0090] Meyers, J. , Luna, T. , & Willon, P. (2020, April 28). Newsom: Reopening California businesses coming soon. *Los Angeles Times*. Retrieved from https://www.latimes.com/california/story/2020-04-28/reopen-california-businesses-gavin-newsom-phases-stay-home-order-coronavirus

[aws21186-bib-0091] Minnesota Department of Health . (2020). *COVID‐19 reopening guidance for noncommunity public water systems*.

[aws21186-bib-0092] Montagnino, E. , Ra, K. , Proctor, C. , & Whelton, A. (2020). Example flushing procedure for a three floor office building. West Lafayette, IN: *Purdue University* .

[aws21186-bib-0093] Muscarella, L. F. (2004). Contribution of tap water and environmental surfaces to nosocomial transmission of antibiotic‐resistant *Pseudomonas aeruginosa* . Infection Control & Hospital Epidemiology, 25, 342–345. 10.1086/502402 15108733

[aws21186-bib-0094] NASEM . (2019). Management of legionella in water systems. Washington, DC: The National Academies Press. 10.17226/25474 32200596

[aws21186-bib-0095] Natural Resources Canada . (2018, May 9). *Recommissioning for existing buildings*. Retrieved from https://www.nrcan.gc.ca/energy/efficiency/energy-efficiency-buildings/energy-efficiency-existing-buildings/recommissioning-existing-buildings/20705

[aws21186-bib-0096] New Zealand Ministry of Business and Environment . (2020). Ensuring the safety of your building water system post covid 19 lockdown water stagnation.

[aws21186-bib-0097] New Zealand Ministry of Health . (2020). COVID‐19 drinking‐water advice returning to normal service.

[aws21186-bib-0098] Nguyen, C. , Elfland, C. , & Edwards, M. (2012). Impact of advanced water conservation features and new copper pipe on rapid chloramine decay and microbial regrowth. Water Research, 46, 611–621. 10.1016/j.watres.2011.11.006 22153355

[aws21186-bib-0099] Niedeveld, C. J. , Pet, F. M. , & Meenhorst, P. L. (1986). Effect of rubbers and their constituents on proliferation of legionella pneumophila in naturally contaminated hot water. The Lancet, 328, 180–184. 10.1016/S0140-6736(86)92486-4 2873437

[aws21186-bib-0100] NY State . (2015). *NY State senate bill S8158*. New your State senate, Albany, NY. Retrieved from https://www.nysenate.gov/legislation/bills/2015/s8158

[aws21186-bib-0101] Ohio Environmental Protection Agency . (2020) *Flushing your home when water service is restored*. Columbus, OH.

[aws21186-bib-0102] Ohio Environmental Protection Agency and Ohio Department of Health . (2020). *Guidance for premise plumbing water service restoration*.

[aws21186-bib-0103] Oklahoma Department of Environmental Quality . (2020). *Water quality recommendations for opening closed or less frequently used buildings*.

[aws21186-bib-0104] Oregon Health Agency Public Health Division . (2020). OHA 2322R guidance for reopening building water systems after prolonged shutdown. Retrieved from http://healthoregon.org/dwp

[aws21186-bib-0105] Ortolano, G. A. , McAlister, M. B. , Angelbeck, J. A. , Schaffer, J. , Russell, R. L. , Maynard, E. , & Wenz, B. (2005). Hospital water point‐of‐use filtration: A complementary strategy to reduce the risk of nosocomial infection. American Journal of Infection Control, 33, S1–S19. 10.1016/j.ajic.2005.03.014 15940112

[aws21186-bib-0106] OSHA . Safety and health topics—legionellosis (legionnaires disease and pontiac fever)—control and prevention—Occupational Safety and Health Administration. Retrieved from https://www.osha.gov/SLTC/legionnairesdisease/control_prevention.html#collapse1

[aws21186-bib-0107] Parkes, L. O. , & Hota, S. S. (2018). Sink‐related outbreaks and mitigation strategies in healthcare facilities. Current Infectious Disease Reports, 20, 42. 10.1007/s11908-018-0648-3 30128678

[aws21186-bib-0108] Parshley, L. (2020, April 3). N95 mask shortage forces health workers to disregard infection control. *Vox*. Retrieved from https://www.vox.com/2020/4/3/21206726/coronavirus-masks-n95-hospitals-health-care-doctors-ppe-shortage

[aws21186-bib-0109] PHE . (2020, March 30). *Public Health England. RE: COVID‐19 and food water and environmental microbiology services*. Retrieved from https://www.cieh.org/media/4103/covid-19-and-food-water-and-environmental-microbiology-services-phe.pdf

[aws21186-bib-0110] PID . (2018) *Paradise Irrigation District (PID) advises bottled water only for drinking*, *cooking and brushing teeth*. Paradise, CA. Retrieved from https://pidwater.com/wqadvisory/101‐paradise‐irrigation‐district‐pid‐advises‐bottled‐water‐only‐for‐drinking‐cooking‐and‐brushing‐teeth

[aws21186-bib-0111] Proctor, C. R. , Lee, J. , Yu, D. , Shah, A. D. , & Whelton, A. J. (2020). Wildfire caused wide‐spread drinking water distribution network contamination. AWWA Water Science. 10.1002/aws2.1183

[aws21186-bib-0112] Proctor, C. R. , Reimann, M. , Vriens, B. , & Hammes, F. (2018). Biofilms in shower hoses. Water Research, 131, 274–286. 10.1016/J.WATRES.2017.12.027 29304381

[aws21186-bib-0113] ProEdge Dental . (2020, April). *Ensuring CDC‐compliant DUWL for COVID19 reopen*. Retrieved from https://cdn2.hubspot.net/hubfs/4740883/COVID19/COVID19ReopenProtocol_4.2020_V3.pdf

[aws21186-bib-0114] PSPC . (2020) *Public services and procurement Canada (PSPC) building water systems minimum requirements‐(COVID‐19)*.

[aws21186-bib-0115] Public Health Madison & Dane County . (2020). Water quality and your business: Tips for re‐opening after closure make sure your building's water system and devices are safe to use.

[aws21186-bib-0116] Ra, K. , Montagnino, E. , Proctor, C. , & Whelton, A. (2020). Example Flushing procedure for a school building. West Lafayette, IN: *Purdue University* .

[aws21186-bib-0117] Raetz, M. A. (2010, August 5). *Lead and copper corrosion control in new construction: shock chlorination*, *flushing to remove debris & in‐line device product testing* (Masters Thesis). Virginia Tech.

[aws21186-bib-0118] Rhoads, W. , Chamber, B. , Pearce, A. , & Edwards, M. (2015). Green building design: Water quality considerations. Water Research Foundation project 4383, Denver, CO.

[aws21186-bib-0119] Rhoads, W. J. , Bradley, T. N. , Mantha, A. , Buttling, L. , Keane, T. , Pruden, A. , & Edwards, M. A. (2020). Residential water heater cleaning and occurrence of legionella in Flint, MI. Water Research, 171, 115439. 10.1016/j.watres.2019.115439 31940510

[aws21186-bib-0120] Rhoads, W. J. , Ji, P. , Pruden, A. , & Edwards, M. A. (2015). Water heater temperature set point and water use patterns influence legionella Pneumophila and associated microorganisms at the tap. Microbiome, 3, 67. 10.1186/s40168-015-0134-1 26627188 PMC4666224

[aws21186-bib-0121] Rhoads, W. J. , Pearce, A. , Pruden, A. , & Edwards, M. A. (2015). Anticipating the effects of Green buildings on water quality and infrastructure. Journal—American Water Works Association., 107, 50–61. 10.5942/jawwa.2015.107.0058

[aws21186-bib-0122] Rhoads, W. J. , Pruden, A. , & Edwards, M. A. (2016). Survey of Green building water systems reveals elevated water age and water quality concerns. Environmental Science: Water Research and Technology., 2, 164–173. 10.1039/c5ew00221d

[aws21186-bib-0123] Rochaway, T. , Wiling, G., & Schreck, R. (2007). Performance of elastomeric components in contact with potable water. *Water Research Foundation Project #2932*. Denver, Colorado.

[aws21186-bib-0124] Sacchetti, R. , De Luca, G. , Guberti, E. , & Zanetti, F. (2015). Quality of drinking water treated at point of use in residential healthcare facilities for the elderly. International Journal of Environmental Research and Public Health, 12, 11163–11177. 10.3390/ijerph120911163 26371025 PMC4586667

[aws21186-bib-0125] Salehi, M. , Abouali, M. , Wang, M. , Zhou, Z. , Nejadhashemi, A. P. , Mitchell, J. , … Whelton, A. J. (2018). Case study: Fixture water use and drinking water quality in a new residential Green building. Chemosphere, 195, 80–89. 10.1016/J.CHEMOSPHERE.2017.11.070 29253792

[aws21186-bib-0126] Salehi, M. , Odimayomi, T. , Ra, K. , Ley, C. , Julien, R. , Nejadhashemi, A. P. , … Whelton, A. (2020). An investigation of spatial and temporal drinking water quality variation in Green residential plumbing. Building and Environment, 169, 106566. 10.1016/j.buildenv.2019.106566

[aws21186-bib-0127] Shaw, E. , Gavaldà, L. , Càmara, J. , Gasull, R. , Gallego, S. , Tubau, F. , … Pujol, M. (2018). Control of endemic multidrug‐resistant gram‐negative bacteria after removal of sinks and implementing a new water‐safe policy in an intensive care unit. Journal of Hospital Infection, 98, 275–281. 10.1016/j.jhin.2017.10.025 29104124

[aws21186-bib-0128] Siebel, E. , Wang, Y. , Egli, T. , & Hammes, F. A. (2016). Correlations between Total cell concentration, Total adenosine tri‐phosphate concentration and heterotrophic plate counts during microbial monitoring of drinking water. Drinking Water Engineering and Science, 1, 1.

[aws21186-bib-0129] Singh, R. , Hamilton, K. A. , Rasheduzzaman, M. , Yang, Z. , Kar, S. , Fasnacht, A. , … Gurian, P. L. (2020). Managing water quality in premise plumbing: Subject matter experts' perspectives and a systematic review of guidance documents. Water (Switzerland)., 12(2), 347. 10.3390/w12020347

[aws21186-bib-0130] Stamm, J. M. , Engelhard, W. E. , & Parsons, J. E. (1969). Microbiological study of water‐softener resins. Applied Microbiology, 18, 376–386 Retrieved from http://www.ncbi.nlm.nih.gov/pubmed/5373675 5373675 10.1128/am.18.3.376-386.1969 PMC377988

[aws21186-bib-0131] Swain, M. , McKinney, E. , & Susskind, L. (2020). Water shutoffs in older American cities: Causes, extent, and remedies. Journal of Planning Education and Research, 0739456X2090443. 10.1177/0739456X20904431

[aws21186-bib-0132] Sydnor, E. R. M. , Bova, G. , Gimburg, A. , Cosgrove, S. E. , Perl, T. M. , & Maragakis, L. L. (2012). Electronic‐eye faucets: Legionella species contamination in healthcare settings. Infection Control & Hospital Epidemiology, 33, 235–240. 10.1086/664047 22314059

[aws21186-bib-0133] Takajo, I. , Iwao, C. , Aratake, M. , Nakayama, Y. , Yamada, A. , Takeda, N. , … Okayama, A. (2019). Pseudo‐outbreak of mycobacterium Paragordonae in a hospital: Possible role of the aerator/rectifier connected to the faucet of the water supply system. Journal of Hospital Infection, 104, 545–551. 10.1016/j.jhin.2019.11.014 31785317

[aws21186-bib-0134] Totaro, M. , Valentini, P. , Costa, A. L. , Giorgi, S. , Casini, B. , & Baggiani, A. (2018). Rate of legionella pneumophila colonization in hospital hot water network after time flow taps installation. Journal of Hospital Infection, 98, 60–63. 10.1016/j.jhin.2017.08.021 28890285

[aws21186-bib-0135] Sonoma State University . (2016). *Drinking water: monitoring program*, *bacteriological site sampling plan & emergency notification plan*. Retrieved from http://library.sonoma.edu/default.php

[aws21186-bib-0136] US EPA . (2016). *Technologies for legionella control in premise plumbing systems: Scientific literature review*.

[aws21186-bib-0137] US EPA Region 8 . (2020, March 20). *Revised total coliform rule seasonal startup checklist*. US EPA. Retrieved from https://www.epa.gov/region8-waterops/revised-total-coliform-rule-seasonal-startup-checklist

[aws21186-bib-0138] USEPA . (2013). Revised total coliform rule (RTCR) 78 FR 10269, February 13, 2013, Vol. 78, No. 30. USA.

[aws21186-bib-0139] USEPA . (2018a) *Prepared for contamination in your distribution system?*

[aws21186-bib-0140] USEPA . (2018b) *Guidance for responding to drinking water contamination incidents disclaimer*. Retrieved from https://www.epa.gov/sites/production/files/2018-12/documents/responding_to_dw_contamination_incidents.pdf

[aws21186-bib-0141] van der Lugt, W. , Euser, S. M. , Bruin, J. P. , Den Boer, J. W. , Walker, J. T. , & Crespi, S. (2017). Growth of legionella Anisa in a model drinking water system to evaluate different shower outlets and the impact of cast iron rust. International Journal of Hygiene and Environmental Health, 220, 1295–1308. 10.1016/j.ijheh.2017.08.005 28869187

[aws21186-bib-0142] Van Hoof, J. , Hornstra, L. M. , Van Der Blom, E. , Nuijten, O. W. , & Van Der Wielen, P. W. (2014). The presence and growth of legionella species in thermostatic shower mixer taps: An exploratory field study. Building Services Engineering Research and Technology, 35, 600–612. 10.1177/0143624414527097

[aws21186-bib-0143] van Ingen, J. , Kohl, T. A. , Kranzer, K. , Hasse, B. , Keller, P. M. , Katarzyna Szafrańska, A. , … Sax, H. (2017). Global outbreak of severe mycobacterium chimaera disease after cardiac surgery: A molecular epidemiological study. The Lancet Infectious Diseases, 17, 1033–1041. 10.1016/S1473-3099(17)30324-9 28711585

[aws21186-bib-0144] Vancouver Coastal Health . (2020). *Attention: Facility owners*, *managers*, *and operators issue: Water stagnation risks due to prolonged reduced building occupancy*.

[aws21186-bib-0145] Verweij, P. E. , Meis, J. F. G. M. , Christmann, V. , Van Der Bor, M. , Melchers, W. J. G. , Hilderink, B. G. M. , & Voss, A. (1998). Nosocomial outbreak of colonization and infection with *Stenotrophomonas maltophilia* in preterm infants associated with contaminated tap water. Epidemiology and Infection, 120, 251–256. 10.1017/S0950268898008735 9692603 PMC2809402

[aws21186-bib-0146] VHA . (2014). Veterans health administration (VHA) directive 1061. Prevention of healthcare‐associated legionella disease and scald injury from potable water distribution systems. pp. 1–4.

[aws21186-bib-0147] Walksi, T. , Chase, D. , Savic, D. , Grayman, W. , Beckwigh, S. , & Koelle, E. (2003). Advanced water distribution modeling and management (1st ed.). Waterbury, CT: Haestad Methods.

[aws21186-bib-0148] Wang, J. L. , Chen, M. L. , Lin, Y. E. , Chang, S. C. , & Chen, Y. C. (2009). Association between contaminated faucets and colonization or infection by nonfermenting gram‐negative bacteria in intensive care units in Taiwan. Journal of Clinical Microbiology, 47, 3226–3230. 10.1128/JCM.00034-09 19587299 PMC2756896

[aws21186-bib-0149] Washington State Department of Health . (2020, April 30). *COVID‐19 guidance for legionella and building water system closures. Version 2*. 2020.

[aws21186-bib-0150] Water Works Association . (2017) Replacement and flushing of lead service lines. ANSI/AWWA C810‐17. *AWWA Standard*. 10.12999/AWWA.C810.17

[aws21186-bib-0151] Weber, D. J. , Rutala, W. A. , Blanchet, C. N. , Jordan, M. , & Gergen, M. F. (1999). Faucet aerators: A source of patient colonization with *Stenotrophomonas maltophilia* . American Journal of Infection Control, 27, 59–63. 10.1016/S0196-6553(99)70077-5 9949380

[aws21186-bib-0152] Whiley, H. (2017). Legionella risk management and control in potable water systems: Argument for the abolishment of routine testing. International Journal of Environmental Research and Public Health, 14(1), 12. 10.3390/ijerph14010012 PMC5295263 28029126

[aws21186-bib-0153] Whiley, H. , Giglio, S. , & Bentham, R. (2015). Opportunistic pathogens *Mycobacterium avium* complex (MAC) and *Legionella* spp. colonise model shower. Pathogens, 4, 590–598. 10.3390/pathogens4030590 26213977 PMC4584274

[aws21186-bib-0154] WHO . (2011). *Water safety in buildings*. World Health Organization.

[aws21186-bib-0155] Xing, Q. , Li, G. , Xing, Y. , Chen, T. , Li, W. , Ni, W. , Deng, K. , Gao, R. , Chen, C. , Gao, Y. , Li, Q. , Yu, G. , Tong, J. , Li, W. , Hao, G. , Sun, Y. , Zhang, A. , Wu, Q. , Li, Z. , & Pan, S. (2020). *Precautions are needed for COVID‐19 patients with coinfection of common respiratory pathogens*. *medRxiv*. Cold Spring Harbor Laboratory Press. 10.1101/2020.02.29.20027698

[aws21186-bib-0156] Zlatanović, L. , van der Hoek, J. P. , & Vreeburg, J. H. G. (2017). An experimental study on the influence of water stagnation and temperature change on water quality in a full‐scale domestic drinking water system. Water Research, 123, 761–772. 10.1016/j.watres.2017.07.019 28732329

