# Supplementary material for: Considerations for large building water quality after extended stagnation
Source: AWWA Water Sci. 2020 Aug 6;2(4):e1186. doi: 10.1002/aws2.1186 (PMC7323006; doi:10.1002/aws2.1186)
Supplement: Supplementary file 1 — Appendix S1. Supporting Information [file AWS2-2-0-s001.docx]

**Supporting Information**

**Considerations for Large Building Water Quality after Extended Stagnation**

*Caitlin R. Proctor^1,¥^, William J Rhoads^2,¥^, Tim Keane^3^, Maryam Salehi^4^, Kerry Hamilton^5^, Kelsey Pieper^6^, David M. Cwiertny^7^, Michele Prévost^8^, Andrew J. Whelton*^,9^*

1. *Purdue University, West Lafayette, IN*
2. *Virginia Tech, Blacksburg, VA*
3. *Legionella Risk Management, Inc., Chalfont, PA*
4. *University of Memphis, Memphis, TN*
5. *Arizona State University, Tempe, AZ*
6. *Northeastern University, Boston, MA*
7. *University of Iowa, Iowa City, IA*
8. *Polytechnique Montreal, Montréal, Québec, Canada*
9. *Purdue University, West Lafayette, IN*

** Corresponding author: Andrew J. Whelton,* [*awhelton@purdue.edu*](mailto:awhelton@purdue.edu)*; T: (765) 494-2160*

**Content**

**SI-1.** Reactions occurring during stagnation

**Figure S1.** Alternative building designs with point-of-use water heating. Top: What occupants can see; Option 1: Traditional trunk-and-branch; Option 2: Trunk-and-branch with headers for every flow; Option 3: Trunk-and-branch with multiple risers. TAKEHOME: What consumers see may look the same, but how it is plumbing should dictate the order and volume flushed during recommissioning. Specific considerations may need to be made for alternative/atypical plumbing components (e.g., rainwater collection systems) or energy- or water-saving devices (e.g., point-of-use water heaters).

**Table S1**. Applicability of documents, standards, and codes referred to in order to development of guidance.

**Table S2:** Guidance developed since COVID-19 for building water management

**SI-2.** Key Messages Regarding Building Water Safety and Anticipated Actions For Stakeholders

**SI-1. Reactions occurring during stagnation occurring during stagnation.**

Several reactions affecting chemical and microbial water quality have been observed in water of plumbing systems. Most of the reactions described here have only been observed on the time-scale of hours to days. It is unknown how reactions will continue on the order of weeks to months. Some reactions may reach a stopping point (e.g., carrying capacity of growth), while others may continue to degrade water quality indefinitely. The reactions presented here pertain to (i) disinfectant residual, (ii) corrosion control, (iii) microbial growth, and (iv) other issues, such as taste, odor, and disinfectant byproduct formation.

Disinfectant residual stability. Disinfectant residuals are used in U.S. drinking water to maintain microbial stability and prevent the microorganism proliferation. For buildings that rely on a public water system, the level and stability of disinfectant residual in water delivered to the POE can be highly variable. This is based on a multitude of factors, including type of disinfectant (free chorine or monochloramine), amount of disinfectant typically maintained in municipal distribution system, the location within the distribution system, pipe materials used, and corrosivity of the water, among others [1–7]. During normal building operation, a disinfectant residual is not always detectable at the building POE: It one study, it was present in only 10% of 58 discrete sampling events in a year [8]. Disinfectant residuals can dissipate rapidly within the plumbing system, especially in hot water systems [8–11]. Residual was found to decay at highly stagnant taps >140 times faster than in corresponding municipal water [12]. These problems may be exacerbated in green buildings that have low water use and do not turn over water stored in the system regularly[8,12,13].

Decreased effectiveness of corrosion control. Plumbing material corrosion is dependent on water chemistry (e.g., dissolved oxygen, pH, disinfection), pipe characteristics (e.g., material, diameters, lengths), temperature, and water flow [14]. Corrosion control treatment aims to reduce the presence of soluble and particulate metals in water primarily by addition of corrosion inhibitors (e.g., phosphates or silicates) and water pH/alkalinity adjustments to promote formation of stable scales. During periods of stagnation, lead and other heavy metals can leach from plumbing materials, and issues are common in homes, schools, and buildings with lead-bearing plumbing (e.g., service line, brass, pre-1986 lead-tin solder). While lead equilibrium is reached fairly rapidly (e.g., ½-¾ inch diameter lead pipes within 24 hrs [15]), redox gradients will develop as a function of water age and can create corrosion “hot spots” [16]. During stagnation, corrosion scales can become destabilized and/or modified, but there has been limited research on prolonged stagnation. Release of other regulated metals (e.g., arsenic) from scales may be a concern. Moreover, researchers have highlighted the dynamic relationship between corrosion and disinfection [17,18]. If corrosion is not controlled, localized issues and damage (e.g., pitting) can occur.

Microbial issues. Microbial growth during water stagnation is well documented [11,19,20], and may result in nitrification, growth of opportunistic pathogens, and community shifts. Nitrifying bacteria, which oxidize ammonia-nitrogen and catalyze residual destruction [21–23] may become more prevalent in distributed and building water due to stagnation, particularly in summer months (i.e., temperature >15 °C, 68 °F). This is a particular problem for chloraminated systems. Nitrification can produce nitrite and nitrate, contaminants with regulated drinking water limit of 1 mg/L and 10 mg/L (measured as N), respectively [24]. These are linked to methemoglobinemia or “blue-baby syndrome”. Some utilities may temporarily convert from chloramine to a free chlorine residual in all or part of the distribution system to remove nitrogen-containing ammonia nutrients and starve nitrifying bacteria [25]. This temporary switch has implications for water corrosivity and measurements taken at the building level to confirm if a disinfectant is present (i.e., free vs total chlorine (Cl_2_)).

Opportunistic pathogens are a significant health concern in building water systems, especially where water can produce aerosols when used. These organisms cause infections primarily in immunocompromised persons after inhalation, aspiration, and/or dermal exposure. *Legionella pneumophila* is linked to issues with inadequate control measures (e.g., temperature too low) in hot water systems, design (e.g., dead ends), decreased effectiveness of corrosion control, and uncontrolled external events in the public water distribution systems and building plumbing [26], which are anticipated to become worse because of stagnation. Several studies have identified growth of *Legionella* during stagnation, although this curve may plateau[27–30]. Other opportunistic pathogens, including non-tuberculous mycobacteria (e.g., *Mycobacterium avium* complex), *Pseudomonas aeruginosa*, and free-living amoeba (e.g., *Acanthomoeba*, *Vermamoeba*) have unique ecological niches (e.g., responsiveness to chlorine vs. chloramine) [31], but are also likely to grow during stagnation [32–34]. To minimize the risk of pathogen growth in plumbing, healthcare facilities routinely flush outlets and POU devices [35–38], maintain growth-deterrents (i.e., hot water; disinfectant residual), and hydraulically balance systems to minimize stagnation [39–43]. Since public water systems can also have opportunistic pathogens, stagnation in the distribution system may pose an additional risk [44–46].

The impact of prolonged stagnation on microbial ecology changes are unclear, but significant microbial community shifts have been observed in stagnant water and stagnant distal ends on short time scales (i.e., days) [11,19,20,47–50]. In particular, biofilm structure is influenced by flow regime [51–53], which changes during stagnation and may have implications for biofilm mobilization, including pathogens, when water use is resumed. Nutrients to sustain growth vary based on water source, treatment methods, and season, and could be introduced during stagnation through low levels of water use, nutrient cycling within biofilms [54], and necrotrophic growth [55].

Other issues. A number of other water quality issues have been documented with stagnation. The concentration of some disinfectant byproducts may increase to unacceptable levels as disinfectant residual is consumed during stagnation time (over several hours), especially with high temperatures [56,57]. As these reactions may occur quickly, the significance of disinfectant byproduct formation during prolonged stagnation is unclear. While many plumbing designs maintain design temperatures (i.e., hot water hot, cold water cold) by insulating pipes and spacing them apart, heat gain in cold water systems and heat loss in hot water systems are inevitable during normal operating conditionos [39]. Ambient indoor temperatures that control temperature in stagnant pipes [58–60] will be dependent on climate and indoor temperature (for pipes in conditioned areas): During closures, these may change relative to normal building operation and may influence corrosion reaction rates and growth conditions. For example, owners of summer homes sometimes drain plumbing to avoid pipe-bursts or freezing in unoccupied and unheated homes during the winter.

1 Hallam, N.B., West, J.R., Forster, C.F., Powell, J.C. and Spencer, I. (2002) The Decay of Chlorine Associated with the Pipe Wall in Water Distribution Systems. *Water Research*. https://doi.org/10.1016/S0043-1354(02)00056-8.

2 Clark, R.M. and Haught, R.C. (2005) Characterizing Pipe Wall Demand: Implications for Water Quality Modeling. *Journal of Water Resources Planning and Management*. https://doi.org/10.1061/(ASCE)0733-9496(2005)131:3(208).

3 Clark, R.M. (2011) Chlorine Fate and Transport in Drinking Water Distribution Systems: Results from Experimental and Modeling Studies. *Frontiers of Earth Science*. https://doi.org/10.1007/s11707-011-0194-x.

4 Munavalli, G.R., Kumar, M.S.M. and Kulkarni, M.A. (2009) Wall Decay of Chlorine in Water Distribution System. *Journal of Water Supply: Research and Technology - AQUA*. https://doi.org/10.2166/aqua.2009.048.

5 Tamminen, S., Ramos, H. and Covas, D. (2008) Water Supply System Performance for Different Pipe Materials Part I: Water Quality Analysis. *Water Resources Management*. https://doi.org/10.1007/s11269-008-9244-x.

6 Masters, S., Wang, H., Pruden, A. and Edwards, M.A. (2015) Redox Gradients in Distribution Systems Influence Water Quality, Corrosion, and Microbial Ecology. *Water Research*. https://doi.org/10.1016/j.watres.2014.09.048.

7 Dias, V.C.F., Besner, M.C. and Prévost, M. (2017) Predicting Water Quality Impact after District Metered Area Implementation in a Full-Scale Drinking Water Distribution System. Journal - American Water Works Association. https://doi.org/10.5942/jawwa.2017.109.0099.

8 Salehi, M., Odimayomi, T., Ra, K., Ley, C., Julien, R., Nejadhashemi, A.P., Hernandez-Suarez, J.S., Mitchell, J., Shah, A.D. and Whelton, A. (2020) An Investigation of Spatial and Temporal Drinking Water Quality Variation in Green Residential Plumbing. *Building and Environment*. https://doi.org/10.1016/j.buildenv.2019.106566.

9 Bédard, E., Laferrière, C., Charron, D., Lalancette, C., Renaud, C., Desmarais, N., Déziel, E. and Prévost, M. (2015) Post-Outbreak Investigation of Pseudomonas Aeruginosa Faucet Contamination by Quantitative Polymerase Chain Reaction and Environmental Factors Affecting Positivity. *Infection Control and Hospital Epidemiology*. https://doi.org/10.1017/ice.2015.168.

10 Charron, D., Bédard, E., Lalancette, C., Laferrière, C. and Prévost, M. (2015) Impact of Electronic Faucets and Water Quality on the Occurrence of Pseudomonas Aeruginosa in Water: A Multi-Hospital Study. *Infection Control and Hospital Epidemiology*. https://doi.org/10.1017/ice.2014.46.

11 Bédard, E., Laferrière, C., Déziel, E. and Prévost, M. (2018) Impact of Stagnation and Sampling Volume on Water Microbial Quality Monitoring in Large Buildings. *PLoS ONE*. https://doi.org/10.1371/journal.pone.0199429.

12 Rhoads, W.J., Pruden, A. and Edwards, M.A. (2016) Survey of Green Building Water Systems Reveals Elevated Water Age and Water Quality Concerns. Environmental Science: Water Research and Technology. https://doi.org/10.1039/c5ew00221d.

13 Rhoads, W.J., Pearce, A., Pruden, A. and Edwards, M.A. (2015) Anticipating the Effects of Green Buildings on Water Quality and Infrastructure. *Journal - American Water Works Association*. https://doi.org/10.5942/jawwa.2015.107.0058.

14 Triantafyllidou, S. and Edwards, M. (2012) Lead (Pb) in Tap Water and in Blood: Implications for Lead Exposure in the United States. Critical Reviews in Environmental Science and Technology. https://doi.org/10.1080/10643389.2011.556556.

15 Schock, M.R. (1989) Understanding Corrosion Control Strategies for Lead. *Journal of the American Water Works Association*. https://doi.org/10.1002/j.1551-8833.1989.tb03244.x.

16 Masters, S., Parks, J., Atassi, A. and Edwards, M.A. (2015) Distribution System Water Age Can Create Premise Plumbing Corrosion Hotspots. *Environmental Monitoring and Assessment*. https://doi.org/10.1007/s10661-015-4747-4.

17 Speth, T., DeSantis, M., Schock, M. and Lytle, D. (2019) Potential Corrosion Issues Resulting from Extreme Weather Events. 13th CECIA-IAUPR Biennial Symposium on Potable Water Issues in Puerto Rico, Bayamon, PUERTO RICO. February 14 - 16, 2019.

18 Rhoads, W.J., Garner, E., Ji, P., Zhu, N., Parks, J., Schwake, D.O., Pruden, A. and Edwards, M.A. (2017) Distribution System Operational Deficiencies Coincide with Reported Legionnaires’ Disease Clusters in Flint, Michigan. *Environmental Science and Technology*. https://doi.org/10.1021/acs.est.7b01589.

19 Ling, F., Whitaker, R., LeChevallier, M.W. and Liu, W.-T. (2018) Drinking Water Microbiome Assembly Induced by Water Stagnation. *The ISME Journal*, Nature Publishing Group, 1. https://doi.org/10.1038/s41396-018-0101-5.

20 Lautenschlager, K., Boon, N., Wang, Y., Egli, T. and Hammes, F. (2010) Overnight Stagnation of Drinking Water in Household Taps Induces Microbial Growth and Changes in Community Composition. *Water Res*, **44**, 4868–4877. https://doi.org/10.1016/j.watres.2010.07.032.

21 Wong-Chong, G.M. and Loehr, R.C. (1975) The Kinetics of Microbial Nitrification. *Water Research*. https://doi.org/10.1016/0043-1354(75)90108-6.

22 Charley, R.C., Hooper, D.G. and McLee, A.G. (1980) Nitrification Kinetics in Activated Sludge at Various Temperatures and Dissolved Oxygen Concentrations. *Water Research*. https://doi.org/10.1016/0043-1354(80)90002-0.

23 WILD, H., SAWYER CN and MCMAHON TC. (1971) Factors Affecting Nitrification Kinetics. *Journal of the Water Pollution Control Federation*.

24 EPA. National Primary Drinking Water Regulations | Ground Water and Drinking Water | US EPA. https://www.epa.gov/ground-water-and-drinking-water/national-primary-drinking-water-regulations.

25 Zhang, Y., Love, N. and Edwards, M. (2009) Nitrification in Drinking Water Systems. Critical Reviews in Environmental Science and Technology. https://doi.org/10.1080/10643380701631739.

26 Scanlon, M.M., Gordon, J.L., McCoy, W.F. and Cain, M.F. (2020) Water Management for Construction: Evidence for Risk Characterization in Community and Healthcare Settings: A Systematic Review. *International journal of environmental research and public health*, NLM (Medline), **17**. https://doi.org/10.3390/ijerph17062168.

27 Rogers, J., Dowsett, A.B., Dennis, P.J., Lee, J. V and Keevil, C.W. (1994) Influence of Temperature and Plumbing Material Selection on Biofilm Formation and Growth of Legionella Pneumophila in a Model Potable Water System Containing Complex Microbial Flora. *Applied and environmental microbiology*, **60**, 1585–92. http://www.ncbi.nlm.nih.gov/pubmed/8017938.

28 Yee, R.B. and Wadowsky, R.M. (1982) Multiplication of Legionella Pneumophila in Unsterilized Tap Water. *Applied and Environmental Microbiology*. https://doi.org/10.1128/aem.43.6.1330-1334.1982.

29 Cooper, I.R., White, J., Mahenthiralingam, E. and Hanlon, G.W. (2008) Long-Term Persistence of a Single Legionella Pneumophila Strain Possessing the Mip Gene in a Municipal Shower despite Repeated Cycles of Chlorination. *The Journal of hospital infection*, **70**, 154–9. https://doi.org/10.1016/j.jhin.2008.06.015.

30 Sharaby, Y., Rodríguez-Martínez, S., Oks, O., Pecellin, M., Mizrahi, H., Peretz, A., Brettar, I., Höfle, M.G. and Halpern, M. (2017) Temperature Dependent Growth Modeling of Environmental and Clinical Legionella Pneumophila Multilocus Variable-Number Tandem-Repeat Analysis (MLVA) Genotypes. *Applied and Environmental Microbiology*. https://doi.org/10.1128/AEM.03295-16.

31 Falkinham, J.O., Hilborn, E.D., Arduino, M.J., Pruden, A. and Edwards, M.A. (2015) Epidemiology and Ecology of Opportunistic Premise Plumbing Pathogens: Legionella Pneumophila, Mycobacterium Avium, and Pseudomonas Aeruginosa. *Environmental health perspectives*, **123**, 749–58. https://doi.org/10.1289/ehp.1408692.

32 Haig, S.J., Kotlarz, N., Lipuma, J.J. and Raskin, L. (2018) A High-Throughput Approach for Identification of Nontuberculous Mycobacteria in Drinking Water Reveals Relationship between Water Age and Mycobacterium Avium. *mBio*. https://doi.org/10.1128/mBio.02354-17.

33 NASEM. (2019) (National Academies of Sciences Engineering and Medicine). Management of Legionella in Water Systems. Washington, DC: The National Academies Press. https://doi.org/10.17226/25474.

34 Bédard, E., Prévost, M. and Déziel, E. (2016) Pseudomonas Aeruginosa in Premise Plumbing of Large Buildings. MicrobiologyOpen. https://doi.org/10.1002/mbo3.391.

35 Garrison, L.E., Kunz, J.M., Cooley, L.A., Moore, M.R., Lucas, C., Schrag, S., Sarisky, J. and Whitney, C.G. (2016) Vital Signs: Deficiencies in Environmental Control Identified in Outbreaks of Legionnaires’ Disease-North America, 2000-2014. *Morbidity and Mortality Weekly Report*. https://doi.org/10.15585/mmwr.mm6522e1.

36 Marciano-Cabral, F., MacLean, R., Mensah, A. and LaPat-Polasko, L. (2003) Identification of Naegleria Fowleri in Domestic Water Sources by Nested PCR. *Applied and Environmental Microbiology*. https://doi.org/10.1128/AEM.69.10.5864-5869.2003.

37 Bartram, J., Chartier, Y., Lee, J. V., Pond, K. and Surman-Lee, S. (2007) Legionella and the Prevention of Leginoellosis. *World Health Organization*.

38 Ciesielski, C.A., Blaser, M.J. and Wang, W.L.L. (1984) Role of Stagnation and Obstruction of Water Flow in Isolation of Legionella Pneumophila from Hospital Plumbing. *Applied and Environmental Microbiology*. https://doi.org/10.1128/aem.48.5.984-987.1984.

39 Bédard, E., Fey, S., Charron, D., Lalancette, C., Cantin, P., Dolcé, P., Laferrière, C., Déziel, E. and Prévost, M. (2015) Temperature Diagnostic to Identify High Risk Areas and Optimize Legionella Pneumophila Surveillance in Hot Water Distribution Systems. *Water Research*, **71**, 244–256. https://doi.org/10.1016/j.watres.2015.01.006.

40 Rhoads, W.J., Ji, P., Pruden, A. and Edwards, M.A. (2015) Water Heater Temperature Set Point and Water Use Patterns Influence Legionella Pneumophila and Associated Microorganisms at the Tap. *Microbiome*, **3**, 67. https://doi.org/10.1186/s40168-015-0134-1.

41 Darelid, J., Löfgren, S. and Malmvall, B.E. (2002) Control of Nosocomial Legionnaires’ Disease by Keeping the Circulating Hot Water Temperature above 55°C: Experience from a 10-Year Surveillance Programme in a District General Hospital. *Journal of Hospital Infection*. https://doi.org/10.1053/jhin.2002.1185.

42 Liu, Z., Lin, Y.E., Stout, J.E., Hwang, C.C., Vidic, R.D. and Yu, V.L. (2006) Effect of Flow Regimes on the Presence of Legionella within the Biofilm of a Model Plumbing System. *Journal of Applied Microbiology*. https://doi.org/10.1111/j.1365-2672.2006.02970.x.

43 Boppe, I., Bédard, E., Taillandier, C., Lecellier, D., Nantel-Gauvin, M.A., Villion, M., Laferrière, C. and Prévost, M. (2016) Investigative Approach to Improve Hot Water System Hydraulics through Temperature Monitoring to Reduce Building Environmental Quality Hazard Associated to Legionella. *Building and Environment*. https://doi.org/10.1016/j.buildenv.2016.08.038.

44 LeChevallier, M.W. (2019) Occurrence of Culturable *Legionella Pneumophila* in Drinking Water Distribution Systems. *AWWA Water Science*, Wiley, **1**, e1139. https://doi.org/10.1002/aws2.1139.

45 Pierre, D., Baron, J.L., Ma, X., Sidari, F.P., Wagener, M.M. and Stout, J.E. (2019) Water Quality as a Predictor of Legionella Positivity of Building Water Systems. *Pathogens*. https://doi.org/10.3390/pathogens8040295.

46 Keane, T. (2012) Case Studies of Legionnaires’ Disease Outbreaks Related to Municipal Water Disruptions. AWWA WQTC Toronto.

47 Ji, P., Parks, J., Edwards, M.A. and Pruden, A. (2015) Impact of Water Chemistry, Pipe Material and Stagnation on the Building Plumbing Microbiome. *PloS one*, Public Library of Science, **10**, e0141087. https://doi.org/10.1371/journal.pone.0141087.

48 Proctor, C.R., Gächter, M., Kötzsch, S., Rölli, F., Sigrist, R., Walser, J.-C. and Hammes, F. (2016) Biofilms in Shower Hoses – Choice of Pipe Material Influences Bacterial Growth and Communities. *Environ. Sci.: Water Res. Technol.*, The Royal Society of Chemistry, **2**, 670–682. https://doi.org/10.1039/C6EW00016A.

49 Inkinen, J., Jayaprakash, B., Santo Domingo, J.W., Keinänen-Toivola, M.M., Ryu, H. and Pitkänen, T. (2016) Diversity of Ribosomal 16S DNA- and RNA-Based Bacterial Community in an Office Building Drinking Water System. *Journal of Applied Microbiology*, **120**, 1723–1738. https://doi.org/10.1111/jam.13144.

50 Proctor, C.R., Reimann, M., Vriens, B. and Hammes, F. (2018) Biofilms in Shower Hoses. *Water Research*, Pergamon, **131**, 274–286. https://doi.org/10.1016/J.WATRES.2017.12.027.

51 Fish, K., Osborn, A.M. and Boxall, J.B. (2017) Biofilm Structures (EPS and Bacterial Communities) in Drinking Water Distribution Systems Are Conditioned by Hydraulics and Influence Discolouration. *Science of the Total Environment*. https://doi.org/10.1016/j.scitotenv.2017.03.176.

52 Douterelo, I., Sharpe, R.L. and Boxall, J.B. (2013) Influence of Hydraulic Regimes on Bacterial Community Structure and Composition in an Experimental Drinking Water Distribution System. *Water Res*, **47**, 503–516. https://doi.org/10.1016/j.watres.2012.09.053.

53 Manuel, C.M., Nunes, O.C. and Melo, L.F. (2009) Unsteady State Flow and Stagnation in Distribution Systems Affect the Biological Stability of Drinking Water. *Biofouling*, Taylor & Francis, **26**, 129–139. https://doi.org/10.1080/08927010903383448.

54 Pruden, A. and Wang, H. FIGURE 3-1 The Influence of Water Chemistry and Flow on the Microbiome of Bulk Water Pipes. as Cited in National Academies of Sciences, Engineering, and Medicine. 2017. *Microbiomes of the Built Environment: A Research Agenda for Indoor Microbiology, Human Health, and Buildings. Washington, DC: The National Academies Press*.

55 Temmerman, R., Vervaeren, H., Noseda, B., Boon, N. and Verstraete, W. (2006) Necrotrophic Growth of Legionella Pneumophila. *Applied and environmental microbiology*, American Society for Microbiology, **72**, 4323–8. https://doi.org/10.1128/AEM.00070-06.

56 Dion-Fortier, A., Rodriguez, M.J., Sérodes, J. and Proulx, F. (2009) Impact of Water Stagnation in Residential Cold and Hot Water Plumbing on Concentrations of Trihalomethanes and Haloacetic Acids. *Water Research*. https://doi.org/10.1016/j.watres.2009.04.019.

57 Liu, B. and Reckhow, D.A. (2015) Impact of Water Heaters on the Formation of Disinfection By-Products. Journal - American Water Works Association. https://doi.org/10.5942/jawwa.2015.107.0080.

58 Salehi, M., Abouali, M., Wang, M., Zhou, Z., Nejadhashemi, A.P., Mitchell, J., Caskey, S. and Whelton, A.J. (2018) Case Study: Fixture Water Use and Drinking Water Quality in a New Residential Green Building. *Chemosphere*, Pergamon, **195**, 80–89. https://doi.org/10.1016/J.CHEMOSPHERE.2017.11.070.

59 Zhang, H.-H., Chen, S.-N., Huang, T.-L., Shang, P.-L., Yang, X. and Ma, W.-X. (2015) Indoor Heating Drives Water Bacterial Growth and Community Metabolic Profile Changes in Building Tap Pipes during the Winter Season. *International journal of environmental research and public health*, Multidisciplinary Digital Publishing Institute (MDPI), **12**, 13649–61. https://doi.org/10.3390/ijerph121013649.

60 Zlatanović, L., Moerman, A., van der Hoek, J.P., Vreeburg, J. and Blokker, M. (2017) Development and Validation of a Drinking Water Temperature Model in Domestic Drinking Water Supply Systems. *Urban Water Journal*, Taylor & Francis, 1–7. https://doi.org/10.1080/1573062X.2017.1325501.


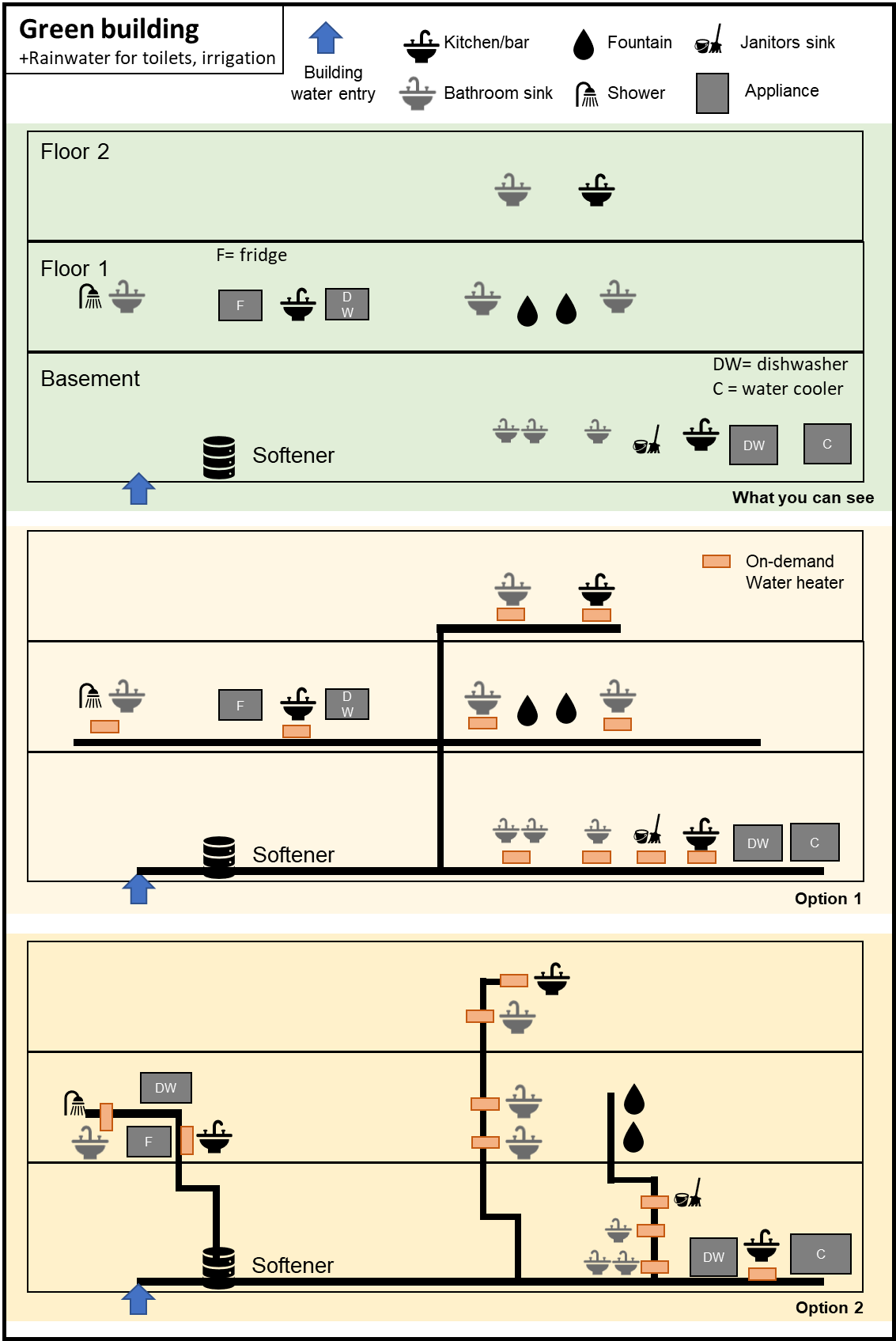


**Figure S1.** Alternative building designs with point-of-use water heating. Top: What occupants can see; Option 1: Traditional trunk-and-branch; Option 2: Trunk-and-branch with headers for every flow; Option 3: Trunk-and-branch with multiple risers. TAKEHOME: What consumers see may look the same, but how it is plumbing should dictate the order and volume flushed during recommissioning. Specific considerations may need to be made for alternative/atypical plumbing components (e.g., rainwater collection systems) or energy- or water-saving devices (e.g., point-of-use water heaters).

**Table S1**: Applicability of documents, standards, and codes referred to in order to development of guidance.

| **Documents, standards, and codes** | **Intended application of code** | | | **Specific health risks explicitly identified** |
| --- | --- | --- | --- | --- |
|  | **Design use** | ****why it is applicable*** | ****why it's not*** |  |
| **UPC (2018): Chapter 6 Water Supply and Distribution** | Govern the materials, design, and installation of water supply systems, including methods and devices used for backflow prevention. | Pertains to preventing health risks | Pertains to new construction | No |
| **IPC (2018): Chapter 6 Water Supply and Distribution** | Govern the materials, design and installation of water supply systems, both hot and cold, for utilization | Pertains to preventing health risks | Pertains to new construction | No |
| **Revised Total Coliform Rule Checklist for Seasonal Potable Water Systems (2016)** | Seasonal public water systems (e.g., campgrounds, fair grounds) | Seasonal use means several months stagnation. | Typically outdoor/buried water systems. Sometimes systems are drained. Rules apply differently if systems remain pressurized. | Indirectly "coliform bacteria" |
| **AWWA 651: Disinfecting Water Mains** | Procedures for the disinfection of new and repaired potable water mains. New water mains shall be disinfected before they are placed in service. Water mains taken out of service for inspection, repair, or other activities may or may not require disinfection and sampling, depending on the risk of contamination. This standard describes the process for evaluating the risk under different conditions. | Pertains to preventing health risks | Pertains to PWS water mains 4-in diam and greater with little to no variety in plumbing materials. Not recommended for plumbing with smaller pipes and a variety of materials | Indirectly "coliform bacteria" |
| **AWWA 652: Disinfecting Water Storage Facilities** | Materials, facility preparation, application of disinfectant to interior surfaces of facilities, and sampling and testing for the presence of coliform bacteria; procedures for disinfection for underwater inspection of online, potable water storage facilities, but does not cover the technical aspects of underwater disinfection. All storage facilities taken out of service for inspection, repairing, painting, cleaning, or other activity that might lead to contamination of water shall be disinfected before they are returned to service | Pertains to preventing health risks | Pertains to PWS water storage tanks | Indirectly "coliform bacteria" |
| **WHO (2010): Water Safety in Buildings** | Developing and implementing building water safety plans and provides examples on how these key principles can be applied to buildings. | Pertains to building plumbing | Some plumbing materials may not be resistant to high concentration of chlorine | Chemical, bacteria, opportunistic pathogens |
| **ASHRAE Guideline 12-2000** | *Legionella* prevention and remediation to provide guidance | Pertains to building plumbing design, operation, and maintenance | Only focused on *Legionella* | *Legionella* |
| **UPC (2021): Informative Appendix N** | Inform buildings operators to the temperature growth ranges for *Legionella* and scalding | Pertains to building plumbing design and operation | Only focused on *Legionella* and scalding | *Legionella* and scalding |

**Table S2:** Guidance developed since COVID-19 for building water management

| **Documents Listed in**  **Order of Most Recent Date Issued** | **Specific health risks explicitly identified** | **Action during building closures** | **Actions Suggested Prior to Building Use** | | | | | | |
| --- | --- | --- | --- | --- | --- | --- | --- | --- | --- |
|  |  |  | Inspection | Flushing (amount, speed) | Other cleaning | Shock disinfection | Other step | Worker safety mentioned | Testing |
| **Expert Report (This Study) &**  **Key Messages In SI Section** | *Legionella, mycobacteria, Pseudomonas aeruginosa, and free-living amoeba; high lead and copper concentrations; disinfectant byproducts.* | Flush hot and cold water outlets at least weekly; Consult public health authority | Consult public health authority | Consult public health authority | Consult public health authority | Consult public health authority | Consult public health authority | Exposure to contaminated water, aerosols with pathogens, shock disinfection water, and scalding. | Consult public health authority |
| **Nations and Organizations with Multiple Nations Represented** | | | | | | | | | |
| **PHE (2020): COVID-19 and Food Water and Environmental Microbiology Services** [23] | *Legionella* | Hot and cold water system outlets should be used at least weekly to minimize stagnation; Consider implementing a flushing regime or other measures such as draining the system if it is to remain vacant for long periods. | Recommends reviewing ESGLI (2020) | Recommends reviewing ESGLI (2020) | Recommends reviewing ESGLI (2020) | Recommends reviewing ESGLI (2020) | Recommends reviewing ESGLI (2020) | Recommends reviewing ESGLI (2020) | Recommends reviewing ESGLI (2020) |
| **ESGLI (2020): Guidance for Managing *Legionella* in Building Water Systems during the COVID-19 Pandemic** [26] | *Legionella* | Building closed for <1 month: Inspect the system. Maintain hot water temperatures. Flush hot and cold water weekly. Monitor in-building disinfectant residual levels. | Not mentioned | Flushing to remove biocide from shock disinfection | Follow advice for other additional water systems and equipment. | Carry out a full system disinfection of the cold water system in accordance with their guidance. Provide hot water to hot water tanks and plumbing | Flush cold water through every outlet. Refill and reheat water heater, then flush outlets. | Scalding | Monitor temperature and biocide levels for at least 48 hr. Collect Legionella samples from the sentinel outlets (microbiological samples collected before 48 hr may give false negative results) |
| **CDC (2020): Guidance for Building Water Systems** [24] | *Legionella* and other biofilm-associated bacteria | Develop a water management plan and perform manufacturer maintenance on equipment | Identify slime on decorative water features, hot tubs/spas, cooling towers; Ensure water heater working | Flush hot and cold water through all points of use. Flushing may need to occur in segments. The purpose of building flushing is to replace all water inside building piping with fresh water. Flush until the hot water reaches its maximum temperature. | Follow manufacturer recommendations for draining the water heater after a prolonged period of disuse. Clean all decorative water features. clean fire sprinkler systems, eye wash stations, and safety showers. | Recommended for hot tubs/spas | Regularly check water parameters. Follow water management plan. Contact local water authority. | Not mentioned | Measure disinfectant levels in refilled water features. Request that disinfectant residual entering the building meets expected standards. |
| **Ireland HSA (2020)** | *Legionella* | Train employees with the Legionella control plan, controls and checks to be carried out; Keep flushing outlets, chemical dosing of evaporative cooling systems; conduct risk assessment; small buildings should do extended weekly flushing of all outlets will assist in maintaining microbiological control;  if building shutdown, water systems should be left filled with water and not drained down. With large water systems, residual water or moisture will remain within the system if drained and biofilm can develop where there are pockets of water or high humidity. The water in the system helps to avoid other problems associated with systems drying out, including failure of tank joints and corrosion in metal pipework. | Water systems, which have been shut down, have had low water usage, or modified control regimes during the pandemic, may result in an increased risk of Legionella bacteria being present. Such systems may have been out of use for a significant time and in most cases cannot simply be used straight away. The system may require recommissioning as if new (that is thorough flushing, cleaning and disinfection and/or controlled flushing of outlets such as taps, showers and toilets) prior to return to use and reopening of the building. Risk assessment review and water testing should also be considered as part of the recommissioning plan. The services of a competent person may be required to provide further advice. | | | | | | |
| **Canada PSPC (2020): Building Water System Minimum Requirements** | *Legionella*, lead | Flush >30 minutes at least every 3 days from points of consumption furthest from POE on the top floor (or the longest run furthest from the water entry for single story sites); Flush each hot and cold water risers or main distribution pipes; Weekly, flush kitchen faucets (hot and cold), drinking fountains, washroom faucets (hot and cold), showers (hot and cold) and eyewash stations for 2 minutes; Remove aerators before flushing, rinse before reinstalling. Maintain a log book. Post sign on outlets: Warn that additional flushing is being conducted and occupants should let the water run for 2 minutes before consuming it, and when washing hands (1 min 20 sec), let the water run to help with flushing the system. | FOR REDUCED OCCUPANCY:  Not mentioned  FOR NO OCCUPANCY >1 WEEK:  Provide alternative drinking water source until sampling demonstrates drinking water quality meets Canadian drinking water quality guidelines; Schedule flushing and testing before the building reopens. | FOR REDUCED OCCUPANCY:  Flush all fixtures (hot and cold) in unoccupied areas; Remove aerators before flushing; Clean and reinstall aerators after flushing; Replace filters after flushing; Flush the hot and cold outlets furthest away from the POE for >30 minutes; Test for disinfectant residual at the tap with the longest residence time (point of consumption farthest from the water entry); Flush all water fixtures (hot and cold) and equipment directly connected to the building water system, such as coffee machines, water coolers and ice machines, floor by floor, for 5 minutes each starting at the water fixture closest to the water entry.   FOR NO OCCUPANCY >1 WEEK:  Same approach as above except | Ensure trap seals are maintained; Pour water into floor drains and flushing each sanitary fixture (i.e. toilet, urinal) once a week to maintain trap seals;  If regular maintenance activities are reduced, drain building water systems that are not being used (e.g., landscape irrigation, water reuse, decorative water features) to avoid stagnant water conditions; Follow start-up procedures, manufacturer recommendations and requirements of LBCMP when re-starting systems. | Not mentioned | Not mentioned | Wear appropriate personal protective equipment (PPE); Consult your employer for requirements; Open outlets slowly to avoid splashing and the creation of aerosols. | FOR REDUCED OCCUPANCY:  No testing  FOR NO OCCUPANCY > 1 WEEK:  Sample for microbiological parameters at POE and at the point of consumption (cold water) furthest from the water entry (longest residence time).  Note that microbiological analyses take twenty-four (24) to forty-eight (48) hours to get results.  In the event of a positive microbiological result, action(s) will have to be taken and alternate sources of drinking water will have to be provided to employees. |
| **New Zealand Ministry of Business and Environment (2020): ENSURING THE SAFETY OF YOUR BUILDING WATER SYSTEM POST COVID 19 LOCKDOWN** | Microorganisms and pathogens, such a*s Legionella*; heavy metals. | Not mentioned | Not mentioned | Flush your water system before reopening; Flush all points of use (e.g. showers, sinks, toilets); Procedures will depend on the building and may need to occur in sections (e.g. floors or individual rooms) due to facility size and water pressure. Consideration should be given to local water use restrictions; 1. Remove tap aerators, POU filters and shower hoses where possible; Removal will allow the water flow rate to be faster and limit the amount of sediment trapped during flushing; 2. a) Open all cold water outlets simultaneously to flush the service line and internal pipework, or b) Flush all outlets individually, starting near where the water enters the building and moving systematically through the building to the most distant outlet; Flush all the cold water pipework first, and then the hot water; 3. Run enough water to replace all water inside building piping with fresh water; The required duration will vary based on pipework volume and outlet velocity; 4. Replace all aerators and POU filters and shower hoses; 5. Additional precautions may be warranted if excessive disruption of pipe scale or if concerns about biofilm development; Actions could include use of bottled water, installation of a POU device, or engaging a contractor to thoroughly clean the plumbing system. | Not mentioned | Not mentioned | Floor building floor drains, pour water into the drain to make sure that the trap water seal is fully restored in order to keep sewer gases from entering the building. Trap water seals can be lost due to evaporation within unoccupied buildings.  Building Services (HVAC/fire/electrical/gas systems etc.)  It is recommended that building managers contact their appropriate maintenance providers to ensure buildings are safely recommissioned before occupancy where necessary. | Not mentioned | Not mentioned |
| **New Zealand Ministry of Health (2020): COVID-19 DRINKING-WATER ADVICE Returning to Normal Service** | Not mentioned | Not mentioned | Approach depends on specified types of buildings;  MUNICIPAL WATER SUPPLY:  Prior to flushing contact your local Council as they may need to advise of an appropriate day and time to flush your system so that this does not create demand issues for the whole supply.  STAND ALONE WATER SUPPLY:  Check for any blockages, leaks or breaks in pipes and taps and fix these where required | MUNICIPAL WATER SUPPLY:  Sufficiently flush all of your taps/faucets so that the water is clear or appears normal; flushing may take longer if you have a large premises or there are still water quality issues after flushing has been completed. When flushing, recommended you use an outdoor tap which is at the furthest point from the road and any faucets (or fountains) that you drink from.  STAND ALONE WATER SUPPLY:  Sufficiently flush water so that is clear or appears normal o When flushing water it is recommended you use a tap which is at the furthest point from your source/treatment. | Not mentioned | Not mentioned | STAND ALONE WATER SUPPLY:  Check your water tank that it has enough water; if any observable physical matter is in the tank (i.e. Animals / leaves etc); Check that all localised treatment systems are operating correctly: Pumps are working, Filters are checked and that they are in good condition and operating correctly or have been replaced, UV systems are turned on and working correctly, there’s sufficient chemical stocks for treating water (i.e. chlorine solutions); run building water treatment system prior to returning to normal so that you know the system is working correctly in advance | Not mentioned | Not mentioned |
| **U.S. State Agencies** | | | | | | | | | |
| **Connecticut Department of Public Health (2020): Building Water System Return to Service Guidance** | Changes in temperature, loss of chlorine residual, biofilm/*Legionella* growth, biofilm disruption, and plumbing corrosion resulting in discolored water, odor, lead and/or copper release, and disinfection byproduct formation | Not mentioned | Contact the public water system for assistance. | Calculate the water volume in the building to determine how long to flush water; Remove aerators prior to flushing faucets; Flush unidirectionally, from the service entrance to the periphery of the plumbing system (distal points); Flush zone-by-zone, zones are branches of the building water system with a common source or parts of the building water system served by a common riser; The first zone to flush is the one nearest the building supply. Flush zones progressively outward from the supply. In each zone, flush the cold water plumbing first and hot water second; Flush continuously for >10 minutes, or based on water volume calculations, to flush pipes; Flush drinking fountains for >10 minutes, or based on water volume calculations, to flush pipes; Flush each area in the building individually (e.g. every patient room, restroom, food service area) through to the distal ends; Flush all equipment with water line connections according to manufacturers' instructions; | Clean fixtures including showerheads; inventory filters on equipment to determine if new filters are needed or need to be ordered and replaced; To clear hot water pipes and water heaters of untreated water:  Run hot water only at all faucets and flush until water runs cool or typically for >15 minutes for a typical household 40 gallon hot water tank, >30 minutes for a hot water tank greater than 40 gallons; Consult water system professionals regarding the draining, flushing, or treatment for large capacity hot water tanks/boilers designed to deliver hot water; Remove and clean end-point devices such as faucet aerators and drinking fountain filters; Remember all other water systems in a building, such as water reuse systems, decorative water features such as fountains, and landscape irrigation systems; Follow manufacturer recommendations for disinfecting all water systems after periods of non-use. | Disinfection with concentrated chlorine should be considered when there is a strong reason to believe the building is contaminated with pathogens like *Legionella pneumophila*, and/or the people who use the building are particularly susceptible to infections like Legionnaires’ disease; Disinfectants are dangerous to handle and can cause serious damage to plumbing system components if used improperly. | Consult a building facility engineer about draining in-building reservoirs; Drain, disinfect, flush, and refill water storage tanks according to your water management plan or professional consultant recommendations; Ongoing flushing can repair destabilized scale and control biofilms; Re-stabilizing scale and controlling biofilms is an ongoing process. In the best case, ongoing flushing is conducted for about 12 weeks – the duration needed for protective scale to re-stabilize and for lead borne on particles to be thoroughly washed from the plumbing system as recommended in an industry standard (AWWA) on flushing related to lead; Recommendations for ongoing flushing include: Make sure each POU tap is opened at least once per day. Flush the full building once per week. Full building flushes proceed the same as the initial flush except water tanks do not need to be drained and hot water flushing times are the same as cold water flushing times. Flush the cold and hot water systems separately – cold first and hot second. During flushing, measure the water quality coming into the building and at some taps in the building. Many building operators will not have the equipment or the ability to make measurements. Even if operators cannot measure water quality, they should still flush the building.  CHECK EQUIPMENT/DEVICES: Run water softeners through a regeneration (flush) cycle; Water filters that are commonly used in refrigerators, faucets, and under the sink, etc. are not designed to remove the specific bacteria commonly present in stagnant water lines. If you ran stagnant water through your filter, the filter could be contaminated. Remove and discard water filters, replace with a new filter following flushing. If you cannot flush or run water when filter is removed, replace with new filter after flushing. Follow manufacturer's directions for filter replacement.  Steam Systems: Check if clean steam is used for autoclaves. Check water quality used for steam generation. Check with manufacturer for instructions to bring system back.  Ice Machines: Clean and sanitize per manufacturer's instructions. Flush ice machines by following the manufacturer's instructions, including: Flush the water line to the machine inlet. Close the valve on the water line behind the machine. Disconnect the water line from the machine inlet. Open the valve and run 5 gallons of water through the valve. Dispose of this water. Close the valve. Reconnect the water line to the machine inlet. Open the valve. Replace any filters and sanitize filter holders. Flush the water lines in the machine. Turn on the machine. Make ice for 1 hour and dispose of this ice. Clean and sanitize all parts and surfaces that come in contact with water and ice per the manufacturer’s instructions.  Drinking Fountains / Water Coolers : Run drinking fountains continuously for 5 minutes to flush the system. Replace filter if applicable. | Open outlets slowly to avoid splashing and the creation of aerosols; Wearing personal protection equipment (PPE) including safety goggles, rubber gloves and NIOSH approved N95 facemasks, if available, are recommend best practices; extended time periods of deferred maintenance may present hazards related to electrical systems, HVAC systems, water intrusion, structural components, and other physical hazards during the period of reopening and reoccupation. Workers and building owners/operators should proceed with caution and carry a heightened awareness of these and other potential risks. | All buildings, test for total coliform.  Buildings serving high-risk populations such as nursing homes and health care facilities should consider testing for *Legionella* using a laboratory certified by the state Environment Lab Certification Program at least 14 days prior to re-opening the building.  Water samples for testing should be collected after taking the remedial steps recommended above. |
| **Washington Department of Health (2020) COVID-19 Guidance for Legionella and Building Water System Closures [version 2, April 30, 2020]** [9] **^1^** | *Legionella, Mycobacterium avium,* lead | PREVENTATIVE AND REMEDIAL FLUSHING – describes routine flushing; turn of water heater and maintain chlorine levels in hot and cold pipes | NO – but recommendations requirement measurement of critical items | YES – limited detail on how to achieve; no velocity specified | SOME SPECIFIC ACTIONS | YES – if Legionella testing demonstrates issue | YES – Attempt high temperature in hot system before disinfecting hot | YES – refers to OSHA | LEGIONELLA (only after “sustained” period of low use and after flushing) |
| **Indiana Department of Environmental Management (2020): IDEM Guidance for Flushing Water Systems** | *Legionella,* corrosion issues |  |  | Flush ALL cold water taps for >5 minutes; open ALL of the cold-water ﬁxtures, ﬂush each toilet at least one time; This does include the water in your refrigerator water dispenser; Flushing should begin at the water service entrance and proceed through the building to the end points of the plumbing system in the building(s). Depending on flow and pressure you may need to increase the flushing time to ensure all the stagnant water has been flushed; Once the cold water lines have been flushed begin the hot water ﬂushing procedure by opening the hot water taps in your bathroom(s). Flush ALL hot water taps for 15 minutes including lavatory (sink) ﬁxtures, hot water bath ﬁxtures, and any other hot water ﬁxtures, such as kitchens, wet bars, etc. Depending on the size of the hot water tank you may need to flush longer to ensure the tank has drained and refilled; For draining and cleaning the water heater consult the manufacturer; Flush ALL remaining Appliances and Faucets, Open any remaining ﬁxtures such as hose bibs, external faucets or ﬁxtures not used for drinking for at least ﬁve minutes to ﬁnish the plumbing system ﬂushing. Remove water from other appliances: ice makers, dishwashers, washing machines, humidifiers, CPAP machines, oral, medical or health care devices, baby formula, water filters, water softeners, filters, POU and whole house filter. Remove ice from ice maker bin and discard 2 additional batches of ice. Run empty dishwasher and washing machine once on rinse cycle; Periodic flushing is important except water tanks do not need to be drained and hot water flushing times are the same as cold water flushing times. Consider measuring chlorine residual during flushing. | Some buildings have water treatment systems and all those treatment devices need to be cleaned, flushed and maintained as part of the starting up process. After ﬂushing, your water ﬁlters need to be replaced. If you have any point of entry water treatment system such as a water softener or ﬁlter, please refer to the instruction manual for replacement of the filter. | Not mentioned | Not mentioned | During the initial flushing, wear protective respiratory equipment. | Not mentioned |
| **Indiana State Department oh Health (2020): BUILDING WATER SYSTEM STARTUP GUIDANCE** | Lead, copper, *Legionella* |  | Inspect plumbing; Repair supply piping or drain leaks found; Replace damaged or open plumbing traps; Remove aerators, POU filters, and shower hoses, and ensure motion sensors have been disabled on automatic faucets. ; Check the integrity of the water system by closing all faucets within the system. Shut down any systems that may be feeding the overall water system, such as water softeners or other water conditioning apparatus. Ensure the water heaters are properly maintained; Confirm the temperature is correctly set - generally at a minimum of 120°F; Determine if the manufacturer recommends draining the water heater after a prolonged period of disuse; Turn on the main supply to the building water system. | Follow the flow of water from the main water supply (use the buildings as-built diagrams if available) and begin flushing at the first location within the system to eliminate all sediment and debris sitting within the main supply line. This may require flushing for up to one hour to properly to remove all sediment and debris from the main line; Then begin moving along the water system to each faucet, spigot, and/or outlet throughout the system. Flush cold water first, and then move on to the hot water. Times of flushing may vary depending on the amount of sedimentation detected, but most likely flushing the cold water for 1 minute and the hot for 30 seconds will suffice. During flushing activities observe plumbing fixtures to ensure no leakage. Repair any piping, fixture, or drain leaks found during flushing. If it is a multi-story facility and an as-built is unavailable it is best practice to begin at the lowest level and work your way up. | Not mentioned | Not mentioned | Once testing is completed and/or interventions implemented replace all aerators, point of use filters, shower hoses, and you may initiate motion sensors for automatic faucets. If bacteria sourced contamination is found to be associated with a hot water heater or water softener refer to the manufacturers guidance to clean, disinfect, and recommission those systems, and conduct confirmation sampling once recommissioned. | Not mentioned | Water testing may be needed. Sample at various points in the system starting at POE for the main water supply, and then at near, middle, and far locations from the water supply, which may be faucets or spigots. Collect samples for residual disinfectant (will depend on source water), bacteriological components (including *legionella*), lead, and copper.  If bacteriology is present in the building water system, then disinfection will be needed. Follow guidance for disinfection which is summarized in the points below for both water well and public water supply sources  Municipal Water Source - If the physical structure is served by a municipal water source, then follow the CDC guidance related to specific comprehensive water management programs associated with the use of the building. This may be specific to hotels, healthcare facilities, office buildings, or schools.  If metals (lead or copper) are present in the building water system, then additional flushing may be needed. If levels are not acceptable, then seek guidance from a water treatment specialist to implement interventions to reduce the health risk exposure. |
| **Oklahoma Department of Environmental Quality (2020) Water Quality Recommendations for Opening Closed or Less Frequently Used Buildings** | Lead, copper, disinfection byproducts, Legionella, biofilm-associated bacteria | Create a water management plan or reference it, maintain water heater with high temperatures, flushing, | Make sure all equipment and points of use are clean. Normal maintenance like cleaning, flushing, disinfecting by manufacturers’ advice | Unclear if flushing for during or at opening. Details include flushing in stages, flushing cold until disinfectant residual matches point incoming building, flushing hot until maximum temperature | Per manufactuer’s advice | Not mentioned | Contact utility about disruptions; they may be able to check residual | Not mentioned | After your building’s water system has returned to normal, ensure that the risk of Legionella and biofilm growth is minimized by regularly checking water quality parameters such as temperature, pH, and disinfectant levels. |
| **Oregon Health Agency Public Health Division (2020) Guidance for Reopening Building Water Systems after Prolonged Shutdown** | *Legionella*, harmful bacteria, lead, copper | Flush weekly – considerations expanded in flushing to right, but unclear if it applies for ‘recommissioning’ or otherwise. | Not mentioned | Flushing guidelines (preventative only) include: . In general, flush one area and fixture at a time, starting in the basement and working upward to other floors. 2. Remove aerators and flush cold water first, then hot water. 3. Be sure the hot water heater is set to at least 120 degrees Fahrenheit. 4. Consider collecting and analyzing one or more coliform bacteria samples after flushing. | Not mentioned | Not mentinoed | Mentions CDC and Purdue websites. Also “the service of a licensed plumber may be helpful” | Not mentioned | Consider testing and analyzing coliform after flushing |
| **Arkansas Department of Health Engineering (2020) Flushing Guidance for Buildings with Low Occupancy or No Occupancy During Covid-19** | Microbial, chemical (lead, copper) | Occasionally flush systems during periods of reduced use (full flush before reoccupancy) | check for sediments | Begin at water meter and move outwards, addressing all outlets | Routine maintenance on tanks, devices; draining; clean aerators, clean ice machines | Not mentioned | NO | Not mentioned | Chlorine residual at the furthermost outlet; swimming pool kits mentioned. Bacteriological samples sent to state lab |
| **Public Health Madison & Dane County (2020) Water Quality and Your Business: Tips for Re-opening After Closure Make Sure Your Building's Water System and Devices Are Safe to Use** | Disease causing organisms, like the ones that cause Legionnaire’s Disease; corrosion control impacted | Flushing as part of normal cleaning and maintenance, as well as final flush before official reopen. More flushing is needed the longer its out of service | Not mentioned | Flush 24-48 hours before reopening. Flushing in stages, starting at service lines, including all plumbing, tanks, fixtures, and equipment like ice machines. Flush 10-30 minutes, remove aerators, open all outlets at once, then individually from start of building, cold then hot until max temperature. Manufacturers instructions for devices | Not mentioned | Not mentioned | Capture and reuse water for outdoor use. Facility plan for flushing should not replace a WMP | Not mentioned | Not mentioned |
| **City of Durham (NC) (2020) Flushing Water Systems for Reopening** | Disease causing microorganisms (mentioned LD); Corrosion control can be impacted | Preventative flushing – mentioned 1-time flush may not be sufficient | NO | Flush in the 24 to 48 hours before a building officially reopens | NO | NO | Capture and reuse water for outdoor use | Not mentioned | Not mentioned |
| **Minnesota Department of Health (2020) COVID-19 Reopening Guidance for Noncommunity Public Water Systems (v2)** | *Legionella,* sediment, loss of residual, lead and copper, bacteria in equipment | Flushing as part of reopening plan | YES | YES | YES – well and storage tanks | NO | Not mentioned | Not mentioned | coliforms |
| **Vancouver Coastal Health (2020) Water Stagnation Risks Due to Prolonged Reduced Building Occupancy** | *Legionella pneumophila* | Maintain water above 50 C, flush periodically at peak flow with aerators removed, reduce access to certain elements | Ensure that cooling towers, decorative water features, hot tubs, swimming pools are operational, reassess tanks, humidifier trays, dead legs, ice machines | Not mentioned | Clean pools | Not mentioned | Assess prior to opening – consult water management plan and water system professionals | Not mentioned | Not mentioned |
| **Ohio Environmental Protection Agency and**  **Ohio Department of Health (2020) Water Quality Recommendations for Opening Closed or Less Frequently Used Buildings ^2^** | Metals (lead and copper); Opportunistic pathogens (*Legionella, Pseudomonas,* non-Tuberculosis mycobacteria); organics (disinfection byproducts, trihalomethanes and haloacetic acids) | Water management plans, flushing as a preventative measure | Other hazards – be aware | Time is variable, aerators should be removed, cold and hot water should be addressed, systemic to ensure disinfectant residual, no one standard, directions in appendix for single family home | No | Flush before disinfection, professional should be used | Continuous disinfection (with equipment) is an option | General risks – PPE mentioned. | Flushing can be verified by testing. Testing for contaminants of concern (inorganic testing, bacteriological testing, unsafe metals, microbial pathogens). Pressure, temperature and disinfectant residual during flushing |
| **Nongovernmental Organizations** | | | | | | | | | |
| **ESPRI (2020) v1: Coronavirus Building Flushing Guidance [no differences found in version 2]** [25] **^3^** | Disease causing (specifically *L. pneumophila*) microorganisms  Toxic metals such as lead. Harmful substances such as disinfection byproducts. | Do not turn off water heaters. On-going flushing (see flushing). Create water management plan.  Shock disinfection can be considered. | Inspect mechanical equipment and examine function. | Flush from building entry to periphery of building. Identify all places water is stored in the building. Sketch building design. Flush zone by zone according to design (start with zone nearest the building entry and then move outward). Open faucets in the zone from nearest to entry to furthest and flush for 5 minutes AND cold water temperature at final tap is steady. Do cold water and then hot water. [Other suggestions for homes] | Flush, clean and maintain treatment systems. Clean showerheads, faucets and other fixtures. Flush and clean any water systems that store water. Replace/ maintain POU filters. Aerators and other flow restrictors removed. | Disinfect building water systems with concentrated chlorine If building is occupied by people more susceptible to infections like legionnaires' disease. | Conduct on going flushing for 12 weeks to stabilize lead scales. Once per week. | Concentrated chlorine exposure | Collect water samples for analysis by a qualified laboratory (only recommended for buildings with specific at-risk populations like children in childcare and elderly people). Ongoing testing when flushing. Measure concentration of disinfectant at point of entry and the furthest tap of the cold water system. Recommend against any measurements, other than for chlorine, unless there is a compelling reason and the owner can understand what the results mean and what to do about them. |
| **IAPMO (2020) Rehabilitating Stagnant Building Water Systems** [22] | *Legionella* and other pathogens, corrosion issues, off color and taste | Regular flushing every 3-4 days | Not mentioned | Flushing through all the valves open at once for a minimum of 10 minutes, and every other device for 5 minutes | End-point devices should be cleaned | Follow manufacturer instructions to | Other building water systems, floor drain traps, filters | YES – high levels of pathogens – open valves slowly, PPE | Legionella – if there are high risk populations |
| **American Water (2020) Has your facility been closed for weeks? Flush the Pipes** [23] | Lead, *Legionella* | Flush biweekly while closed and the weekend before opening | Not mentioned | Flush toilets at least twice, faucets 2 minutes, showers 2 minutes, until you can smell the chlorine Replace filter after flush | Not mentioned | Not mentioned | Not mentioned | Not mentioned | Not mentioned |
| ^1^ Paper co-author William Rhoads advised on document, perhaps affecting results ^2^ COVID-19 closures were not specifically mentioned, but COVID-19 is mentioned in an appendix document and the authors indicated this document was written in response to COVID-19 closures. ^3^On May 1 2020, the organization added “Reducing Risk to Staff Flushing Buildings” [41] warning about risk to staff flushing buildings, PPE, and strategies to reduce aerosols. | | | | | | | | | |

**SI-2. KEY MESSAGES REGARDING BUILDING WATER SAFETY AND ANTICIPATED ACTIONS FOR STAKEHOLDERS**

The following information was developed based on the study

and preliminary stakeholder feedback prior to publication.

**1. GENERAL**

1. Due to the coronavirus (COVID-19) pandemic “stay-at-home” orders, commercial buildings may now have reduced or no water use.
2. Water in shutdown or low occupancy buildings will stagnate and get old. This water could become unsafe for use.
3. Harmful organisms like *Legionella* can grow in plumbing if the water stagnates. Unsafe levels of lead and copper can also be found.
4. There are 5.6 million non-residential buildings in the U.S. These include: education facilities such as schools and college buildings, daycares, lodging, offices, places of worship, event venues, retail (i.e., shopping malls), libraries, museums, restaurants, healthcare, and other facilities.
5. Utilities, building owners, and health officials can take several immediate actions to help prevent water from becoming unsafe.
6. When buildings reopen, a process called recommissioning can be performed, in which the plumbing can returned to normal use.
7. Occupancy may not reach 100% immediately – a period of low-use may continue. Actions to return plumbing to normal may need repeating as building occupancy increases.

**2. PRIORITY INTERVENTIONS FOR LOW OR NO BUILDING OCCUPANCY**

**2.1 SHORT-TERM CONSIDERATIONS**

*WATER UTILITIES*

1. Develop communication materials to distribute to building owners to inform them how COVID-19 may impact water quality in the distribution system.
2. Communicate to owners of large buildings about the need to maintain fresh water in plumbing.
3. Consider flushing water mains that serve commercial districts where buildings currently have low occupancy or are not being used.
4. Consider analyzing distribution system disinfectant residual data to identify portions of the system that are under-used to help target flushing. Temporarily expanding monitoring locations may better inform actions.
5. Consider increasing disinfectant residual for at least 30 days during peak of post COVID startups.
6. If the system is on monochloramine consider free chlorine burn for at least 30 days during post COVID startup. Consider maintaining higher than normal free chlorine residual during burn.
7. Ask building owners to report low or no water pressure to you immediately.

*HEALTH OFFICIALS AND DEPARTMENTS*

1. Determine the threshold (i.e., time of building closure) that would prompt the public health authority to require restrictions on water use in buildings with low or no occupancy such as: Handwashing Only, Do Not Drink Water, Do Not Use Water.
2. Prepare communication materials for building owners to distribute to occupants regarding water quality in buildings with low or no occupancy. These may include signs to post at water outlets to alert occupants about the status of the plumbing.
3. Identify and alert regional labs immediately about a potential surge in the need for metals and harmful microorganism analysis.
4. Make certain they possess trained personnel, tools, and resources to assist society with re-occupancy efforts.
5. Prepare to deliver guidance to building owners and managers re-occupying buildings and including in that guidance building water system evaluation . Create a checklist for building water system re-occupancy approvals. (Note: public health will normally get this information from hospital admissions but if a building owner notices cluster absence they should report.)
6. Recommend building owners refresh their plumbing by flushing fixtures to bring in fresh water. This can help prevent harmful organisms from growing in plumbing, and dispose of water with unacceptable levels of lead and copper.
7. Prepare to assist building owners assess potential health risks. This includes determining whether to require water testing, when and to collect water samples to assess human health risks.

*BUILDING OWNERS*

1. Contact their government environmental public health departments for advice about building water safety.
2. Communicate information provided by the health department to your building occupants.
3. Consider refreshing your plumbing by flushing fixtures at least weekly. This new, fresh water can help prevent harmful organisms from growing in plumbing. This can also dispose of water with unacceptable levels of lead and copper.
4. Contact a plumber or engineer for assistance.
5. Report clusters of illness to public health

**2.2 LONGER-TERM CONSIDERATIONS**

*BUILDING OWNERS*

1. Contact government environmental public health departments for advice about building water safety.
2. Consider refreshing your plumbing by flushing fixtures at least weekly as this new, fresh water can help prevent harmful organisms from growing in plumbing. This action can also dispose of water with unacceptable levels of lead and copper.
3. Contact a plumber or engineer for assistance in addition to the health department.
4. Recognize that not all buildings, or even of the same type, will undergo the same process for restoring water quality.
5. Communicate information provided by the health department to your building occupants.
6. Consider development of a water management plan.

*UTILITIES*

1. Prioritize restoring water service to buildings for decommissioned health facilities, clinics, and long-term care facilities, and buildings serving vulnerable populations such as early childhood education facilities.
2. Consider continuing to flush water mains that serve commercial districts where buildings currently have low occupancy or are not being used.
3. Consider analyzing distribution system disinfectant residual data to identify portions of the system that are under-used to help target flushing. Temporarily expanding monitoring locations may better inform actions.
4. Coordinate building recommissioning so low pressure and depressurization do not occur when buildings become reoccupied.
5. Ask building owners to report low or no water pressure to you immediately.

*HEALTH OFFICIALS AND DEPARTMENTS*

1. Prioritize oversight of water being restored to decommissioned health facilities, clinics, and long-term care facilities, and buildings serving vulnerable populations.
2. Notify building owners about the process the public health authority has established for certifying or consenting to plumbing use where there’s been low or no occupancy. Not all buildings, or even of the same type, will undergo the same process for restoring water quality.
3. Determine the threshold that would prompt the public health authority to require recommissioning actions such as flushing, fixture cleaning, disinfection, as well as chemical and microbiological testing.
4. Prepare to deliver guidance to building owners and managers re-occupying buildings. Create a checklist for building water system re-occupancy approvals.
5. Remind regional labs about a potential surge in the need for metals and harmful organism analysis.
6. Reconfirm the Health Department possesses trained personnel, tools, and resources to assist society with re-occupancy efforts.
